# Measuring microRNAs: Comparisons of microarray and quantitative PCR measurements, and of different total RNA prep methods

**DOI:** 10.1186/1472-6750-8-69

**Published:** 2008-09-11

**Authors:** Robert A Ach, Hui Wang, Bo Curry

**Affiliations:** 1 Agilent Laboratories, Agilent Technologies, 5301 Stevens Creek Blvd., Santa Clara, CA 95051, USA

## Abstract

**Background:**

Determining the expression levels of microRNAs (miRNAs) is of great interest to researchers in many areas of biology, given the significant roles these molecules play in cellular regulation. Two common methods for measuring miRNAs in a total RNA sample are microarrays and quantitative RT-PCR (qPCR). To understand the results of studies that use these two different techniques to measure miRNAs, it is important to understand how well the results of these two analysis methods correlate. Since both methods use total RNA as a starting material, it is also critical to understand how measurement of miRNAs might be affected by the particular method of total RNA preparation used.

**Results:**

We measured the expression of 470 human miRNAs in nine human tissues using Agilent microarrays, and compared these results to qPCR profiles of 61 miRNAs in the same tissues. Most expressed miRNAs (53/60) correlated well (R > 0.9) between the two methods. Using spiked-in synthetic miRNAs, we further examined the two miRNAs with the lowest correlations, and found the differences cannot be attributed to differential sensitivity of the two methods. We also tested three widely-used total RNA sample prep methods using miRNA microarrays. We found that while almost all miRNA levels correspond between the three methods, there were a few miRNAs whose levels consistently differed between the different prep techniques when measured by microarray analysis. These differences were corroborated by qPCR measurements.

**Conclusion:**

The correlations between Agilent miRNA microarray results and qPCR results are generally excellent, as are the correlations between different total RNA prep methods. However, there are a few miRNAs whose levels do not correlate between the microarray and qPCR measurements, or between different sample prep methods. Researchers should therefore take care when comparing results obtained using different analysis or sample preparation methods.

## Background

MicroRNAs (miRNAs) are small (~18–24 nucleotides) non-coding RNAs which bind to mRNAs to regulate protein expression, either by blocking translation and/or by promoting degradation of the mRNA target (reviewed in [1-3]), or alternatively by increasing translation [4,5]. They have been found to be involved in numerous functions such as cell fate determination, cell proliferation, cell differentiation, and cell death (reviewed in [6,7]). Profiles of miRNAs in various types of tumors have been shown to contain potential diagnostic and prognostic information (reviewed in [8,9]). The number of known miRNAs has rapidly increased in recent years, and currently there are 722 human miRNA sequences reported in the Sanger Institute's miRNA database release 10.0 (miRBase) [10-12], with potentially many more yet to be reported [13,14].

Several methods for global miRNA profiling are currently in common use. These include quantitative RT-PCR (qPCR) involving stem-loop RT primers combined with TaqMan PCR (Applied Biosystems) analysis [15,16], qPCR with locked nucleic acid primers (Exiqon) [17], qPCR using poly(A) tailing (QIAGEN, Stratagene) [18,19], high-throughput sequencing of small RNA libraries [20], and microarray analysis (for examples, see [21-26]). Typical experimental workflows often involve using different methods of measuring miRNAs at different research stages. For this reason, it is important to know how well the different measurements agree with each other. Several groups have compared microarray profiling results with those obtained by quantitative PCR for either a small number of genes or a small set of samples [21,22,26-30]; however there has been no systematic comparison of larger numbers of miRNAs across a widely diverse range of human tissues using the two methods.

In this study, we compared the relative expression of 61 different miRNAs across nine different human tissues, measured using both Agilent miRNA microarrays and TaqMan qPCR. The Agilent microarray platform features the direct end-labeling and profiling of mature miRNAs from total RNA without any size fractionation or amplification to minimize experimental loss, bias, or variations [31,32]. The labeling reaction is performed under denaturing conditions to provide high labeling yield, minimal sequence bias [26], and consistently reproducible efficiency for every miRNA sequence [31,32]. By incorporating hairpin structures in the microarray probe, base-pairing with the additional nucleotide incorporated during labeling, and empirical melting point-determination, the platform is capable of single-nucleotide discrimination in the miRNA sequences while specifically distinguishing the mature miRNAs from longer RNAs in the total RNA sample [26,31,32]. We chose to compare this microarray system against the Taqman qPCR system in particular, since at the time this work was performed this was the most commonly utilized miRNA qPCR system. We found excellent correlation between the microarray and PCR results for most of the miRNAs. We further examined two of the miRNAs showing low correlations by using spiked-in synthetic RNAs, and found that differential sensitivity between the two techniques is not the cause of the discrepancy.

Another factor which could potentially affect the results of an miRNA profiling study is the method used to isolate RNA from the biological sample. Both the Agilent microarray system and the TaqMan qPCR systems use total RNA as the starting material; however, it is unclear whether different total RNA preparation methods will yield systematically different miRNA profiling results. In this report, we compared the results of miRNA microarray profiling obtained with three different commonly used total RNA prep methods. We found that the results for most miRNAs were equivalent among the different sample preparation methods, but that measured levels of a small number of miRNAs differed systematically.

## Results and discussion

### Quantitative RT-PCR and Agilent microarray miRNA profiles correlate strongly

We previously reported that a comparison of Agilent microarray profiling and SYBR green-based quantitative RT-PCR (qPCR) of ten miRNAs in seven different human tissues found the two measurements correlated quite well [26]. To perform a more extensive comparison, we analyzed the expression of 61 human miRNAs in nine different tissues (brain, breast, heart, liver, placenta, testes, ovary, skeletal muscle, thymus), using both Agilent miRNA microarrays and TaqMan stem-loop qRT-PCR [15]. Aliquots of the same RNA samples were used for both the microarray and qPCR measurements. We chose these particular 61 miRNAs for several reasons. First, they represent a wide range of expression levels, as determined in an initial array analysis of some of the tissues. Second, they have wide differences in GC content, ranging from 23% (miR-190) to 68% (miR-328). Third, we chose several miRNAs which had potentially problematic sequences or exhibited atypical behavior during the development of the Agilent microarray platform: two of these did not show as good a linear titration curve as other miRNAs tested in a previous study (miR-126*, miR-296) [26], and two other miRNAs were previously reported not to be labeled by enzymatic methods similar (but not identical) to that used with the Agilent microarray assay (miR-208, miR-219) [33].

Of the 61 miRNAs examined, only miR-637 was not detected by either method in any of the tissues. The rest of the miRNAs assayed were detected in most or all of the tissues by both methods, with two exceptions: miR-208, expressed only in the heart [34] and at very low levels in skeletal muscle, and miR-138, expressed in the brain, and at lower levels in placenta and thymus (all data is shown in Additional File 1).

**Figure 1:**
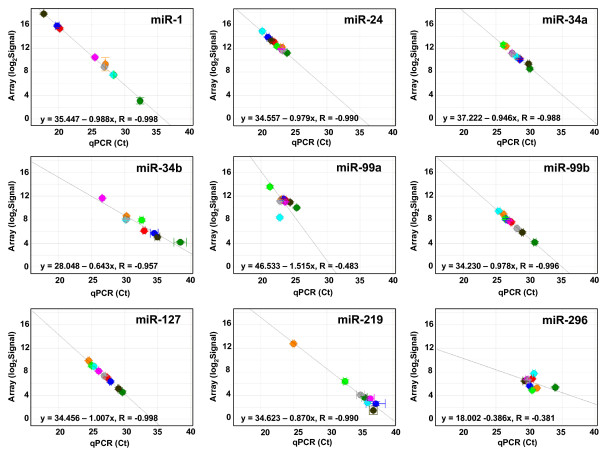
**Comparison of qPCR and microarray miRNA profiling for individual miRNAs in nine human tissues**. Scatter plots are shown for 9 of the 61 miRNAs assayed, with qPCR results (cycle threshold (Ct) values) on the x axes and microarray results (log_2 _of the total gene signal) on the y axes. Each data point represents one tissue. All plots are drawn to the same scale. The equations and R values on each plot are for the orthogonally-fitted line. Spot colors indicate the tissue: red = breast, pink = testes, dark blue = heart, light blue = placenta, dark green = liver, light green = ovary, orange = brain, brown = skeletal muscle, and grey = thymus. Tissues where qPCR results were flagged as "undetermined" by ABI software, or where log2 of the total gene signal on arrays was < 1, were not plotted. Error bars indicate standard deviation (SD) of Ct values for qPCR results and (SD/Mean)*log_2_e of the signals for the array results. Scatter plots for the remaining 51 miRNAs (one miRNA gave no signals with either qPCR or arrays) are in Additional Files 2, 3, 4, 5, 6, 7.

**Figure 2:**
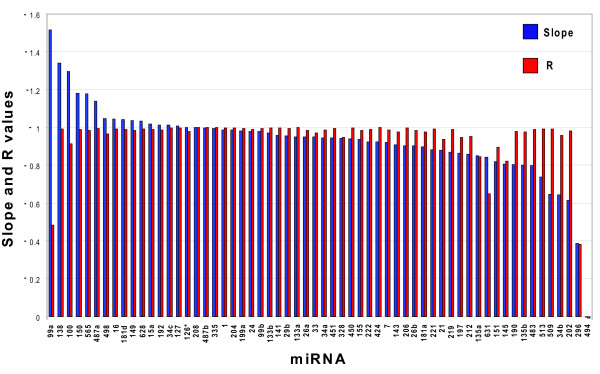
**Slope and R values for all 60 miRNA scatter plots**. The slopes and R values for the orthogonal fit lines for all 60 of the miRNAs plotted in Figure 1 and Additional Files 2, 3, 4, 5, 6, 7 are ordered by the slope values. Slopes are shown in blue and R values are in red.

The qPCR and microarray results were compared by plotting the qPCR cycle threshold (Ct) value versus the log_2 _of the array signal for each miRNA in all nine tissues (representative plots are shown in Figure 1, with the remaining plots shown in Additional Files 2, 3, 4, 5, 6, 7). These two values should be directly comparable, since both the qPCR Ct value and the log_2 _of the microarray signal change by a value of 1 for every 2-fold change in miRNA concentration. If the qPCR and microarray measurements are equivalent, the plots will show a linear correlation (R = -1) with a slope of -1. Figure 2 shows the slopes and the correlation values for each of the 60 miRNAs. 56 of the 60 miRNAs show correlation values (R) between -0.8 and -1.0, and 50/60 plots have slopes between -1.2 and -0.8. Of the four miRNAs which were selected as potentially problematic in the microarray measurements, only miR-296 did not correlate between the microarray and qPCR assays; miR-208, miR-219, and miR-126* all gave excellent correlations.

**Figure 3:**
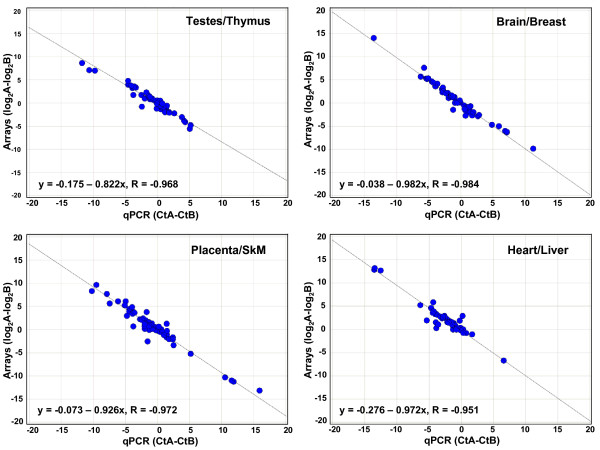
**Comparison of qPCR and microarray miRNA measurements for 60 miRNAs in four tissue pairs**. Scatter plots are shown for miRNA expression ratios in four different tissue pairs, as determined by qPCR (x axis) and microarrays (y axis), where each data point represents one miRNA. The qPCR values are the difference between the Ct values from the two tissues, and the microarray values are the difference between the log_2_(total gene signals) from the two tissues. The equations and R values on each plot are for the orthogonally-fitted line. Scatter plots for the remaining 32 tissue pairs are shown in Additional Files 8, 9, 10, 11, 12, 13, 14, 15. SkM = Skeletal Muscle.

**Figure 4:**
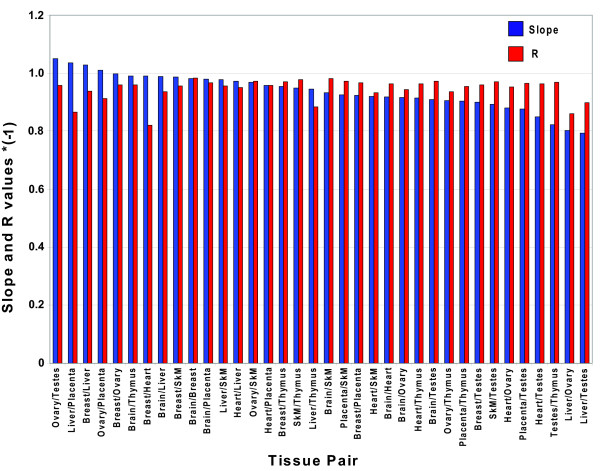
**Slope and R values for all 36 tissue pair plots**. The slopes and R values for the orthogonal fit lines for all 36 possible tissue pairs plotted in Figure 3 and Additional Files 8, 9, 10, 11, 12, 13, 14, 15 are ordered by the slope values. Slopes are shown in blue and R values are in red.

To examine the results for all 60 miRNAs on one plot, we cannot simply plot qPCR Ct values versus microarray signals for all miRNAs in all tissues, because both the qPCR and microarray assays have differential sensitivities to different miRNAs. Thus, instead of looking at absolute expression levels, we must look at relative ratios of miRNA expression between two different tissues. To judge the consistency of fold-changes measured by microarray and qPCR platforms, we plotted the ratios of miRNA expression between all 36 possible pairs of tissues as measured by qPCR (Ct(tissue1)-Ct(tissue2)) and by microarrays (log_2_(signal in tissue1)-log_2_(signal in tissue2)). Four such plots are shown in Figure 3 (the other 32 plots are shown in Additional Files 8, 9, 10, 11, 12, 13, 14, 15), while Figure 4 shows the slopes and R values for each of the plots for the 36 tissue pairs. The plots all show very good correlation between the qPCR and array ratios, with R values between -0.984 and -0.821. The slopes of the 36 plots vary between -1.05 and -0.793. The intercepts of these fold-change plots (shown in Figures 3 and Additional Files 8, 9, 10, 11, 12, 13, 14, 15) indicate the consistency between fold-changes measured by the two methods. The mean of the intercepts of the line fits for the 36 tissue pairs was 0.00 +/- 0.23 (1 SD) (data not shown). This level of variability (17%) is comparable to that seen among independent measurements using the same technique.

### Measurements of spike-ins of miRNAs which systematically differ between platforms show linear sensitivity

**Figure 5:**
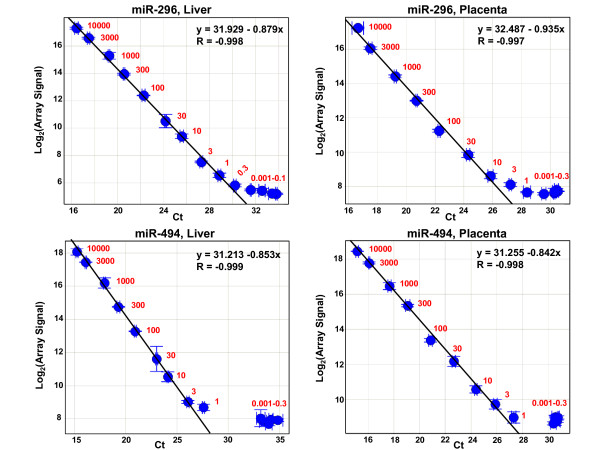
**Comparison of qPCR and microarray measurements for miR-296 and miR-494 titrations**. Scatter plots are shown for titration of synthetic miR-296 into liver (top left panel) and placenta (top right panel) total RNAs, and miR-494 into liver (lower left) and placenta (lower right) total RNAs. Ct values from qPCR are plotted on the x-axis, while log_2 _of the total gene signal from microarray measurements are plotted on the y-axis. Numbers in red show the number of attomoles of spike-in miRNA per 100 ng total RNA. Equations and R values are for the orthogonal line fit of the linear regions of each titration. Error bars indicate standard deviation (SD) of Ct values for qPCR results and (SD/Mean)*log_2_e for the array results.

miR-494, miR-296, and miR-99a are the three miRNAs that exhibit the most discrepant correlation values and slopes between the qPCR and microarray assays (Fig. 2); however, if the measurement of miR-99a in placenta is omitted, the slope for this miRNA becomes -0.844 with R = -0.929 (see plot in Fig. 1). For miR-494 and miR-296, if one platform were measuring levels of these miRNAs accurately, while the other platform were not, then we might expect a significant divergence from linearity to be observed between the two measurements when adding increasing amounts of synthetic miR-494 or miR-296 RNA into a total RNA sample. To test this, we added 1 zmol to 10 fmols of synthetic miR-296 and miR-494 RNAs to 100 ng of total RNA from liver or placenta, and measured the qPCR and microarray responses (Figure 5). For both miRNAs, in both tissues, the relation between qPCR measurement and array measurement is linear above a threshold spike-in concentration. The R values of the linear regions are very close to -1, with slopes between -0.842 and -0.935, indicating that both the qPCR and the microarrays are producing sample-responsive and internally consistent measurements of miR-296 and miR-494 at these concentration levels. Below the threshold spike-in levels, the qPCR Ct values and microarray signals are unchanged for miR-494, while for miR-296 the Ct values increase slightly, but the array measurements are unchanged. We conclude that the difference between the two platforms is not due to different sensitivity, since both the microarray and qPCR measurements are capable of measuring miR-296 and miR-494 accurately above a spike-in concentration threshold. Presumably some type of interference confounds the measurement of endogenous expression levels in the complex sample, on one or both of the platforms.

### Three different total RNA preparation methods show similar yields and quality

A question which arises when comparing the miRNA profiling results reported in different studies is whether the methods used to isolate RNA from tissue or cell line samples systematically affect the miRNA profiles. To examine whether the miRNA profile of a sample is affected by the type of total RNA prep method used, we prepared one large frozen cell pellet from each of two different human cell lines, HeLa (a cervical carcinoma line) and ZR-75-1 (a breast carcinoma line). We then subdivided these pellets into equal aliquots, and performed total RNA isolation on the aliquots using three different techniques: phenol/guanidinium (TRIzol, Invitrogen) followed by isopropanol precipitation, and two column-based techniques, miRNeasy (QIAGEN) and *mir*Vana (ABI). Four to eleven replicate preps were performed on each cell type with each method.

Mean RNA yields, as measured by absorbance at 260 nm, and quality metrics for each prep type are shown in Table 1. The RNA integrities of the preps were analyzed on the Agilent 2100 Bioanalyzer, and all the preps had high quality RNA according to the RIN number [35,36]. This indicates that most of the RNAs in the various preps were intact, with minimal breakdown. However, RIN values do not provide information about non-RNA contaminants, such as organic reagents and DNA. The TRIzol preps showed the lowest mean 260/230 ratios, possibly indicating the presence of some remaining TRIzol reagent in the final product.

**Table 1:** List and characteristics of sample preps from HeLa and ZR-75-1 cell pellets.

**Cell Line**	**Prep Type**	**No. of Preps**	**RNA Yield Mean (SD)**	**260:280 Mean (SD)**	**260:230 Mean (SD)**	**RIN Mean (SD)**
HeLa	TRIzol	10	45.59 (7.17)	1.89 (0.0406)	1.28 (0.208)	9.9 (0.14)
HeLa	*mir*Vana	8	43.16 (12.01)	1.96 (0.0364)	1.36 (0.323)	9.9 (0.11)
HeLa	miRNeasy	11	34.07 (5.40)	2.08 (0.00874)	1.97 (0.389)	9.9 (0.11)
ZR-75-1	TRIzol	4	23.52 (1.39)	1.84 (0.0432)	0.815 (0.0759)	9.8 (0.075)
ZR-75-1	*mir*Vana	4	16.92 (5.84)	2.01 (0.0356)	1.28 (0.361)	9.1 (0.17)
ZR-75-1	miRNeasy	4	18.37 (1.93)	2.05 (0.0311)	1.87 (0.337)	9.8 (0.050)

The number of individual preps performed using the indicated total RNA prep method and cell lines are shown, as are the mean and standard deviations (in parentheses) of the total RNA yields (in micrograms), 260:280 ratios, 260:230 ratios, and RIN numbers for all the preps done with the same method in each cell type. Preps started with either 5 × 10^6 ^cells (HeLa) or 1 × 10^7 ^cells (ZR-75-1).

**Figure 6:**
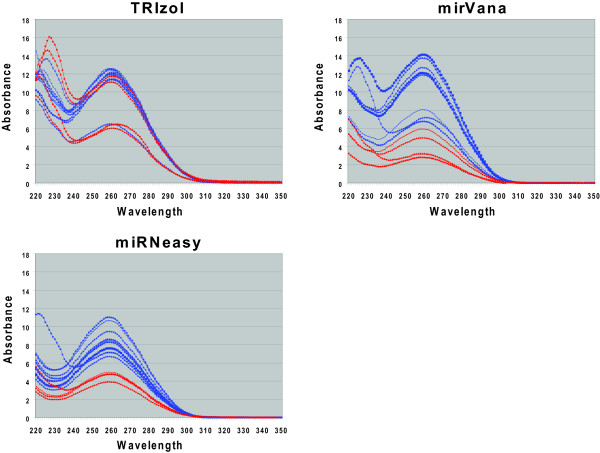
**Absorption Spectra of HeLa and ZR-75-1 Sample Preps**. The absorption spectra for the total RNA sample preps (listed in Table 1) are shown. Blue traces are HeLa cell preps and red traces are ZR-75-1 cell preps. Spectra are from 220 to 350 nm.

Since the absorbance at 260 nm is used to quantitate the amount of RNA for use in the measurement assays, and since the 260:230 ratios can only serve as a crude guideline to possible contaminants, it is important to examine the absorption spectra in more detail (Figure 6). Some of the spectra clearly show the presence of additional peaks between 220–230 nm, indicating the presence of contaminant(s). While this is most consistently seen in the TRIzol preps, it is also sometimes seen in the *mir*Vana preps and in one of the miRNeasy preps. In some of the samples where no distinct peak is observed between 220–230 nm, the spectra show significant absorbance in the wavelengths immediately below 220 nm, with a shoulder tailing from 220 nm to 240 nm. At 240 nm the absorption increases again to peak at around 260 nm, which is the wavelength of maximum absorption for nucleic acids of mixed oligonucleotide composition. For many of these spectra, this absorption pattern suggests that the absorbance of the contaminant(s) whose peak is below 240 nm may overlap with the nucleic acid absorbance peak at 260 nm, which would result in the overestimation of nucleic acid quantity as determined by the absorption at 260 nm. Also, a couple of the absorbance spectra show a slight shoulder in the 260–270 nm range, indicating a contaminant which could also affect RNA quantitation. Thus, careful examination of sample spectra can be important for identifying samples where measured miRNA levels might be compromised by absorbance-based RNA quantitation artifacts.

### Variability of hybridization results is highest between different prep methods

For miRNA microarray profiling analysis we took three of the replicate total RNA preps of each different prep method in each cell type and hybridized them to Agilent miRNA microarrays. A total of 42 hybridizations were done, with all but two samples hybridized at least twice (Additional File 16).

A good measure of the reproducibility of replicate measurements is the Root Mean Square (RMS) deviation of the natural logs of all signals that are well above background levels. The RMS deviation is approximately equal to the coefficient of variation of signals, and is an estimate of the proportional error of the measurement. For example, if the RMS deviation is 0.15 (15%), then a measured fold change between samples of 1.15 is a difference of one standard deviation.

**Figure 7:**
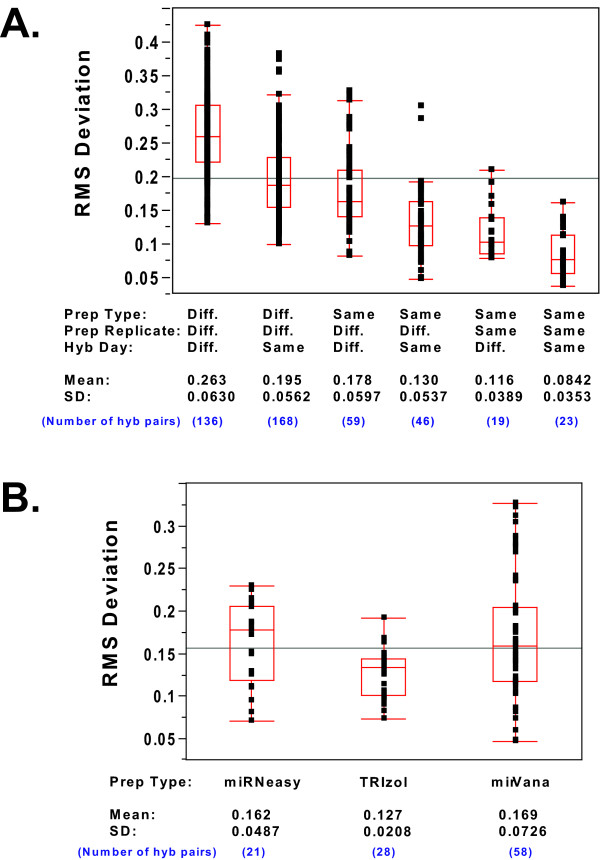
**Box plots of RMS deviations for microarray hybridization pairs**. **(a) RMS deviations of all possible hybridization pairs of the same cell type**. Box plots show the distribution of RMS deviations calculated from all possible pairs of hybridizations done with the same cell type, and sorted by prep type, prep replicate, and hybridization day. Plots show the combined results from HeLa and ZR-75-1 cells. Each replicate pair RMS deviation is indicated by a black dot. The ends of the box represent the 25^th ^and 75^th ^quartiles, while the line through the center of the box represents the median. The whiskers from each box extend to the outermost data point included in the range from the upper quartile plus 1.5*(interquartile range) to the lower quartile minus 1.5*(interquartile range). The line across the entire plot indicates the mean of all the values. Values of the mean and standard deviations for each set of values are shown at the bottom of the plot. **(b) RMS deviations of all prep replicate hybridization pairs, sorted by prep type**. Box plots of the RMS deviations for all the pair-wise comparisons of hybridizations using replicates of the same prep type, and sorted by prep type. Plots show the combined results from HeLa and ZR-75-1 cells. Box plot details are as in Figure 7a.

We compared the RMS deviations between pairs of hybridizations performed using the same total RNA prep (hybridization replicates) with those performed using different total RNA preps done with the same method (prep replicates), and also with those using different total RNA prep methods. We also compared hybridizations performed on the same or different days, in order to take into account any day-to-day variability in the results. Figure 7a shows box plots of the RMS deviations between all possible replicate hybridization pairs, categorized by same or different prep method, same or different prep replicate, same or different hybridization replicate, and same or different hybridization day. The box plots for the six different categories are shown in decreasing order of variability. Hybridizations using preps from different methods show the most variability, with same day hybs of the different prep methods showing slightly less variability than different day hybs. Replicate preparations using the same method are the next lowest in variability, again with same day hybs being less variable than different day hybs. Finally, hybs done with aliquots of the same preparation have the lowest variability, again with same day hybs being less variable than different day hybs. The three sources of variability can thus be put in order of their magnitude: different prep methods > different preps using the same method > different hybridization day.

We also plotted the RMS deviations for hybridizations involving prep replicate pairs from each of the three different prep methods (regardless of the hybridization day), in order to examine whether the different prep methods showed different amounts of variation between prep replicates (Figure 7b). The TRIzol prep replicates showed less variability than the other two prep methods. A Student's t-test between pair-wise comparisons of the three prep methods confirmed that this difference is statistically significant (data not shown). There was no significant difference in variability between the mirVana and miRNeasy prep replicates.

### A small subset of miRNAs differ between prep methods

To examine whether there are systematic differences among the miRNA profiles observed for RNA isolated by different methods, we first looked at the overall signal levels of the hybridizations. The grand means of the mean total gene signal for all hybridizations of the same prep type for each cell line are shown in Table 2. For both cell types, the miRNeasy preps gave about 25% higher overall signals than the other two prep methods. The overall signal differences between the TRIzol and *mir*Vana preps of the same cell type were minimal. It should be noted that the miRNeasy preps had the highest 260:230 ratios (Table 1), and their spectra generally showed lower absorbance in the 220–230 nm range compared to the other two prep types. It is possible that some of the material which is absorbing at 220–230 nm in RNA extracted using the other two methods is contributing to the 260 nm peak, and it is also possible that DNA contaminants are present. The presence of either or both types of contaminant can lead to an overestimation of the amount of RNA present in these preps.

**Table 2:** Grand means of the mean total gene signal for all hybridizations of each prep type.

**Cell Line**	**Prep Type**	**Mean TGS (SD)**
HeLa	TRIzol	235.0 (19.1)
HeLa	*mir*Vana	211.2 (25.8)
HeLa	miRNeasy	290.3 (19.2)
ZR-75-1	TRIzol	407.9 (33.0)
ZR-75-1	*mir*Vana	437.1 (84.5)
ZR-75-1	miRNeasy	546.7 (41.9)

The mean of the total gene signal for all the miRNAs on the microarray were calculated for each individual hybridization, and the mean and standard deviation of these for each prep type in each cell line are shown.

**Figure 8:**
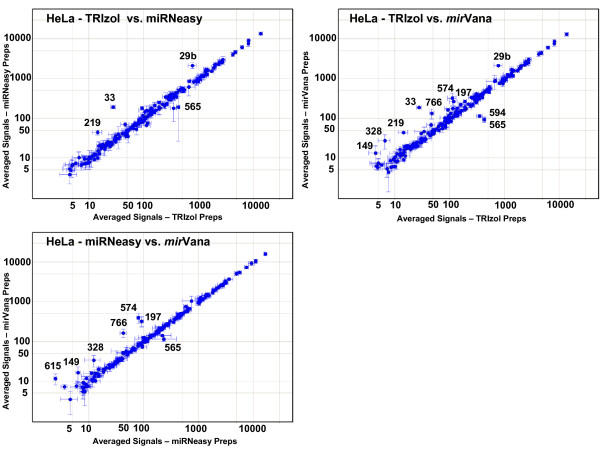
**Pair-wise comparisons of averaged profiles of the three different prep types: HeLa cells**. Total gene signals for each miRNA for all hybridizations of the same RNA prep (hybridization replicates) were averaged, and then these averaged individual prep profiles for all the preps of the same prep type were averaged together to get a mean profile for each of the three prep types. Scatter plots show these averaged profiles from one prep type plotted against another for HeLa cells. Error bars indicate one standard deviation. Numbers indicate the identity of all miRNAs whose signal strengths are at least two-fold higher in one prep type than another, after normalization.

**Figure 9:**
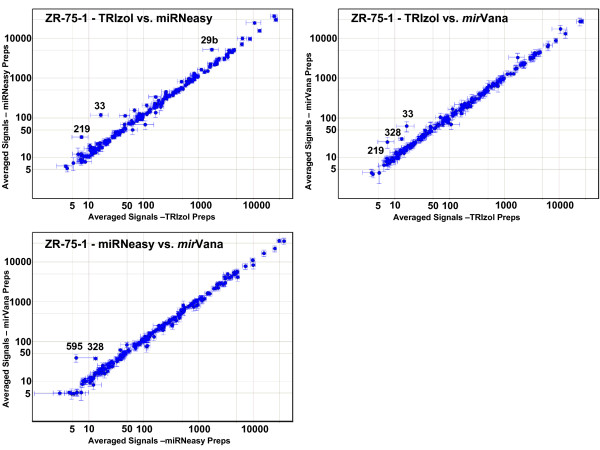
**Pair-wise comparisons of averaged profiles of the three different prep types: ZR-75-1 preps**. Scatter plots as in Figure 8, but for ZR-75-1 cell preps.

We next examined whether specific miRNAs systematically differ among the different prep methods (Figures 8 and 9). We calculated an average expression profile for each prep method for the two cell lines, by first averaging the total gene signals for each miRNA from all hybridizations of the same RNA prep, and then averaging together these individual prep averages for all preps of the same prep and cell type. Since there were differences seen in the overall signal levels between the different preps (Table 2), we normalized each pair-wise comparison to the 75^th ^percentile of one of the pairs. The expression profile of most miRNAs in HeLa cells does not depend on the RNA prep method (Figure 8). However, there is a small subset of miRNAs that consistently report different relative expression levels depending on the prep method. The miRNAs that are labeled in the figure show expression levels that differ by at least 2-fold in different prep methods. Additional File 17 lists the miRNAs which are 1.5x and 2x higher in one prep type compared to another. Three miRNAs are found at consistently lower levels in TRIzol preps than in the other two preps: miR-29b, miR-33, and miR-219. *mir*Vana preps show consistently higher levels of four miRNAs when compared to the other two HeLa preps: miR-149, miR-328, miR-574, and miR-766. Figure 9 (and Additional File 17) shows the results from the ZR-75-1 breast cell line. While fewer miRNAs show different profiles among the three different prep methods in this cell line compared to HeLa, four out of the five that are observed to be discrepant in ZR-75-1 cells are the same as those seen in HeLa cells (miR-29b, miR-33, miR-219, and miR-328).

**Figure 10:**
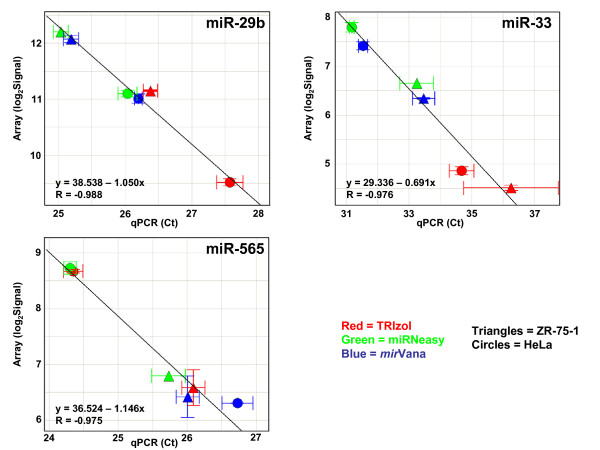
**Comparison of qPCR and microarray miRNA results for three miRNAs differentially measured between different prep types**. Individual TRIzol, miRNeasy, and *mir*Vana preps were assayed with qPCR for three miRNAs found in microarray studies to be at higher levels in one prep type than another. Scatter plots show qPCR results (cycle threshold (Ct) values) on the x axes and microarray results (log_2 _of the total gene signal) on the y axes. Each data point represents one individual prep from one cell type. Circles indicate HeLa preps and triangles represent ZR-75-1 preps. TRIzol preps are in red, miRNeasy preps are in green, and *mir*Vana preps are in blue. The equations and R values on each plot are for the line of best fit. Error bars indicate standard deviation (SD) of Ct values for qPCR results and (SD/Mean)*log_2_e for the array results. Note that the axes are not on the same scale in the three different plots.

The finding that a small number of miRNAs report different microarray signals when prepared by different methods raises the question of whether these differences reflect real differences in the concentrations of these miRNAs in the different sample preps. To examine this, we assayed individual samples prepared with the three methods by qPCR, using primers for three of the miRNAs showing differences between the prep methods. We then compared these results to those obtained from microarray analysis of the same preps (Figure 10). The good agreement of the qPCR results with the array results for these miRNAs strongly suggests that the differences in the miRNA levels observed between the sample prep methods reflect true differences in the miRNA content of the extracted RNA, and are not artifacts of the measurement assay. At present, we have no explanation for why these particular miRNAs are found at different levels when using different extraction techniques.

## Conclusion

In this study we compared the expression levels of 61 miRNAs in nine human tissues as measured by both Agilent microarrays and TaqMan qRT-PCR. We found that 53/60 expressed miRNAs had correlations (R) > 0.9 between the two methods. For the two miRNAs that differed most between the two methods, spike-in studies found the differences are not due to differential sensitivity of the two methods, but are more likely due to interference from other RNAs in the complex mixture.

We also examined microarray-based miRNA profiles using three different total RNA sample prep methods. We found that while almost all miRNA levels correspond between the three different prep methods, a small subset of 2–10 miRNAs consistently differ by greater than 2-fold between different techniques. These differences were corroborated using qPCR, and are most likely due to true differences in the miRNA content of the extracted RNA. Thus, while all three methods are suitable for use in profiling miRNAs from total RNA, it may be prudent to pick one method and use it for the entire course of any particular study, in order to avoid these small profile differences due to the RNA preparation method.

## Methods

### Total RNA and cell samples

Total RNA samples from normal human tissues were from Ambion (Austin, TX). Frozen HeLa cell pellets were from Cell Trends (Middletown, MD), and frozen ZR-75-1 cell pellets were from BioProcessing Inc. (Portland, ME).

### miRNA microarray analysis

miRNA microarrays were manufactured by Agilent Technologies (Santa Clara, CA)., and contain 20–40 features targeting each of 470 human miRNAs (Agilent design IDs 015508 (sample prep studies) and 016436 (nine tissue comparison studies)) [37]. Sequences of the 470 miRNAs were obtained from the Sanger miRBase, release 9.1 [10-12]. Labeling and hybridization of total RNA samples were performed according to the manufacturer's protocol. 100 ng total RNA was used as input into the labeling reaction, and the entire reaction was hybridized to the array for 20 hours at 55°C. For the microarray versus qRT-PCR comparisons, the labeling and hybridizations of the nine human tissues were done 4–5 times, and the mean and standard deviation for each miRNA were calculated.

Microarray results were extracted using Agilent Feature Extraction software (v9.5.3.1) and analyzed using GeneSpring GX 7.3.1 software (Agilent Technologies) and Spotfire DecisionSite 8.1 software (TIBCO Software, Palo Alto, CA). Box plots were calculated using JMP 5.1 software (SAS, Cary, NC). Original microarray data is deposited in the Gene Expression Omnibus [38] (Series GSE11879).

All scatter plots of miRNA microarray data use the total gene signal, which is proportional to the total number of targets bound by the probes targeting each miRNA [31,32]. For comparison of two hybridizations, the natural logs of the total gene signals for all genes expressing above 10x the background noise in both samples were regressed against each other, and the standard deviation of the residuals from the regression line were reported as the RMS deviation. For most pairs of samples prepared by the same method, residuals were normally distributed, so that the RMS deviation describes true random variation in the assay. In pairs of samples prepared by different methods, residuals of most of the miRNAs were also normally distributed, with systematic exceptions of some miRNAs as discussed in the text. No normalization was performed for either microarray or qPCR data, except for an overall intensity normalization applied to the average signals from different prep methods, as described in the text (Figures 8 and 9). For this comparison, the 75^th ^percentile of the total gene signal for all the miRNAs on the array was calculated by sorting the total gene signals for 470 miRNAs on the array in ascending order, and the signals from the three methods were normalized to the signal from the 353rd miRNA.

### miRNA qRT-PCR analysis

miRNA qRT-PCR analysis was performed using Taqman miRNA assays (Applied Biosystems, Foster City, CA), according to the manufacturer's protocol. 5 ng total RNA was input into each reverse transcription reaction (RT) for each miRNA. Four replicates were done for each miRNA, consisting of two replicate PCR reactions from each of the two replicate RT reactions, and the results were averaged. PCR reactions were run on a 7500 Real Time PCR machine (Applied Biosystems) and analyzed using 7500 System SDS software (v1.4).

### miRNA spike-ins

Synthetic miRNAs were manufactured by TriLink BioTechnologies (San Diego, CA) and spiked into human liver and placenta total RNA (Ambion). 100 ng of these RNA mixes were then used for labeling and hybridization onto the microarrays, while 5 ng were used as input into the reverse transcriptase reaction for qPCR. Two replicate microarray hybridizations and four replicate qPCR reactions were done for each dilution in each tissue.

### Total RNA sample preps

Frozen cell pellets were resuspended in phosphate buffered saline and divided into equal aliquots of 5 × 10^6 ^(HeLa) or 1 × 10^7 ^(breast) cells and refrozen. Individual aliquots were subsequently thawed just before use.

TRIzol preps were performed according to the manufacturer's protocol (Invitrogen, Carlsbad, CA) using an isopropanol precipitation. Briefly, 1 ml of TRIzol reagent was added to the cell pellet and cells were lysed by repetitive pipetting, and then incubated at room temperature for 5 minutes. 200 μl of chloroform were added, followed by vigorous shaking and incubation for 2–3 minutes at room temperature. Samples were centrifuged 15 minutes at 12000 × g at 4°C. The aqueous layer was transferred to a new tube, and the RNA was precipitated by adding 0.5 ml isopropanol, incubating 10 minutes at room temperature, and spinning for 10 minutes (12000 × g at 4°C). Pellets were washed with 80% ethanol and resuspended in nuclease-free dH_2_O (Ambion).

miRNeasy total RNA preps (QIAGEN, Valencia, CA) were performed according to the manufacturer's protocol. The *mir*Vana miRNA Isolation kit (Applied Biosystems) was used according to the manufacturer's protocol for total RNA isolation.

All total RNA preps were analyzed using the 2100 Bioanalyzer (Agilent Technologies), RNA 6000 Nano LabChip kits, and 2100 expert software (version B.02.05.SI360). Absorption spectra were measured on an ND-1000 spectrophotometer (NanoDrop Technologies, Wilmington, DE).

## Authors' contributions

RAA helped design the experiments, performed all the experimental work, analyzed the data, and drafted the manuscript. HW and BC helped to design the experiments and to analyze and interpret the data. All authors read and approved the final manuscript.

## Supplementary Material

Additional File 1 **qRT-PCR Ct data and microarray signal data for 61 miRNAs**. Mean Ct values data for the four qPCR replicates are listed, as are mean microarray data (log_2 _of the total gene signal) for the microarray replicates. Click here for file

Additional File 2 **Comparison of qPCR and microarray miRNA profiling for individual miRNAs**. Scatter plots for 51 miRNAs not shown in Figure 1. Click here for file

Additional File 3 **Comparison of qPCR and microarray miRNA profiling for individual miRNAs**. Scatter plots for 51 miRNAs not shown in Figure 1. Click here for file

Additional File 4 **Comparison of qPCR and microarray miRNA profiling for individual miRNAs**. Scatter plots for 51 miRNAs not shown in Figure 1. Click here for file

Additional File 5 **Comparison of qPCR and microarray miRNA profiling for individual miRNAs**. Scatter plots for 51 miRNAs not shown in Figure 1. Click here for file

Additional File 6 **Comparison of qPCR and microarray miRNA profiling for individual miRNAs**. Scatter plots for 51 miRNAs not shown in Figure 1. Click here for file

Additional File 7 **Comparison of qPCR and microarray miRNA profiling for individual miRNAs**. Scatter plots for 51 miRNAs not shown in Figure 1. Click here for file

Additional File 8 **Comparison of qPCR and microarray miRNA profiling for 60 miRNAs in tissue pairs**. Scatter plots for 32 tissue pairs not shown in Figure 3. Click here for file

Additional File 9 **Comparison of qPCR and microarray miRNA profiling for 60 miRNAs in tissue pairs**. Scatter plots for 32 tissue pairs not shown in Figure 3. Click here for file

Additional File 10 **Comparison of qPCR and microarray miRNA profiling for 60 miRNAs in tissue pairs**. Scatter plots for 32 tissue pairs not shown in Figure 3. Click here for file

Additional File 11 **Comparison of qPCR and microarray miRNA profiling for 60 miRNAs in tissue pairs**. Scatter plots for 32 tissue pairs not shown in Figure 3. Click here for file

Additional File 12 **Comparison of qPCR and microarray miRNA profiling for 60 miRNAs in tissue pairs**. Scatter plots for 32 tissue pairs not shown in Figure 3. Click here for file

Additional File 13 **Comparison of qPCR and microarray miRNA profiling for 60 miRNAs in tissue pairs**. Scatter plots for 32 tissue pairs not shown in Figure 3. Click here for file

Additional File 14 **Comparison of qPCR and microarray miRNA profiling for 60 miRNAs in tissue pairs**. Scatter plots for 32 tissue pairs not shown in Figure 3. Click here for file

Additional File 15 **Comparison of qPCR and microarray miRNA profiling for 60 miRNAs in tissue pairs**. Scatter plots for 32 tissue pairs not shown in Figure 3. Click here for file

Additional File 16 **List of microarray hybridizations done with HeLa and ZR-75-1 preps**. Three or four preps done with each method (labeled A-D) were hybridized for the indicated number of times. Click here for file

Additional File 17 **List of miRNAs measured at higher levels in one prep type over another**. miRNAs found at levels either > 2x or between 1.5x and 2x in one prep type over another, in HeLa and ZR-75-1 total RNA preps. Ratios are the ratio of normalized total gene signals on microarrays in prep 1 versus prep 2. Click here for file
