# Supplementary figures and images for: Measuring microRNAs: Comparisons of microarray and quantitative PCR measurements, and of different total RNA prep methods
Source: BMC Biotechnol. 2008 Sep 11;8:69. doi: 10.1186/1472-6750-8-69 (PMC2547107; doi:10.1186/1472-6750-8-69)

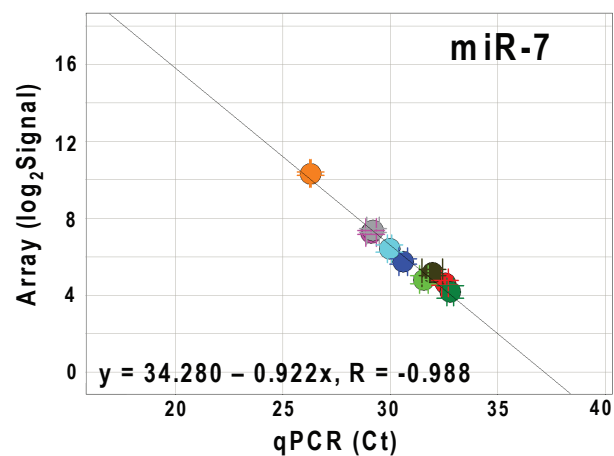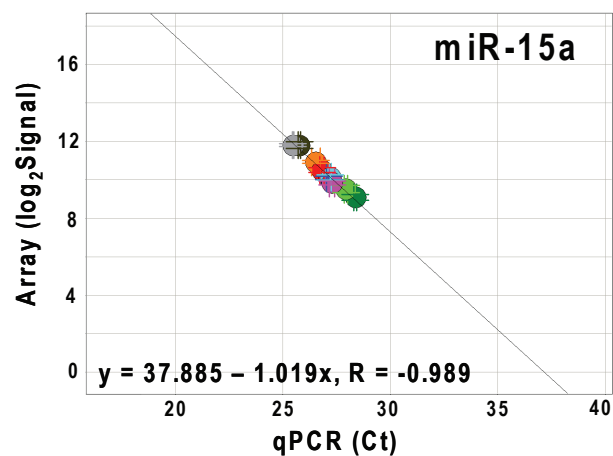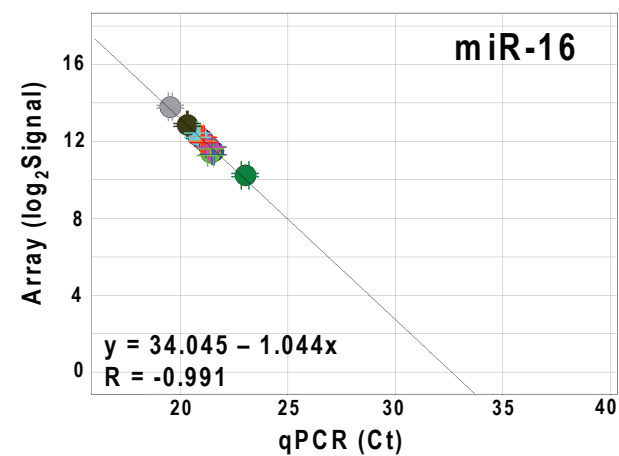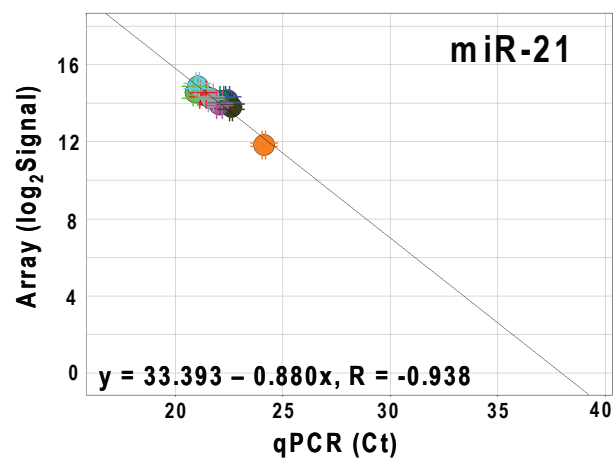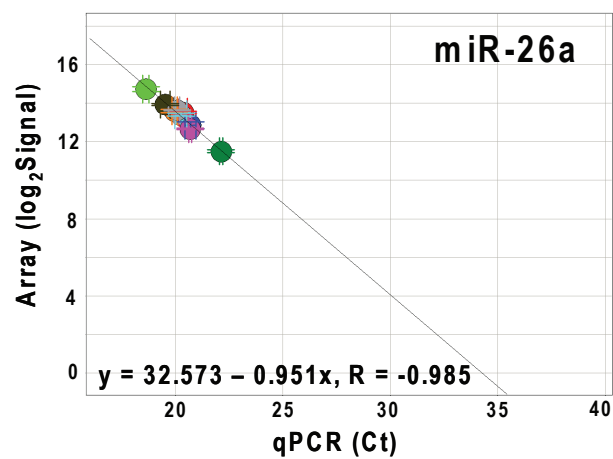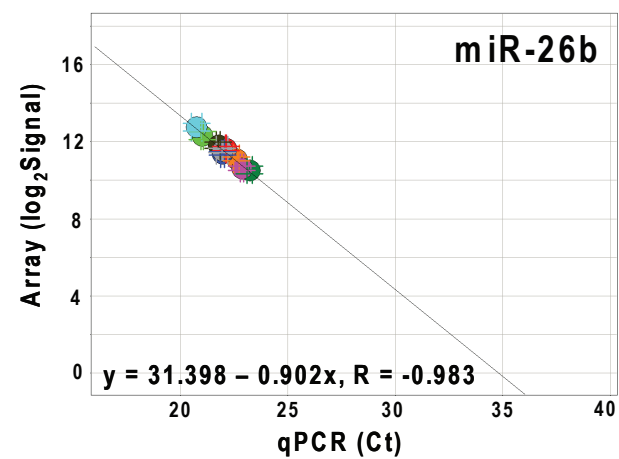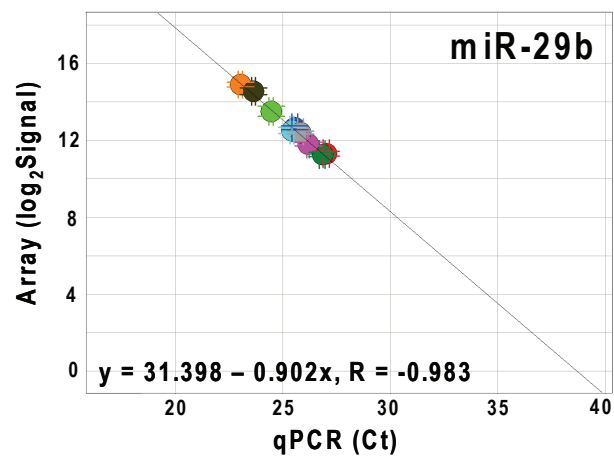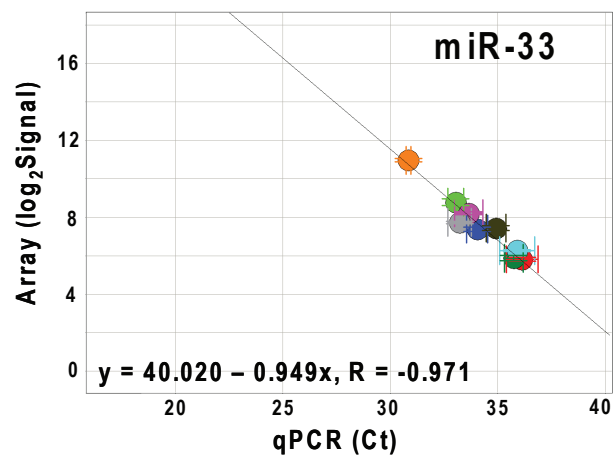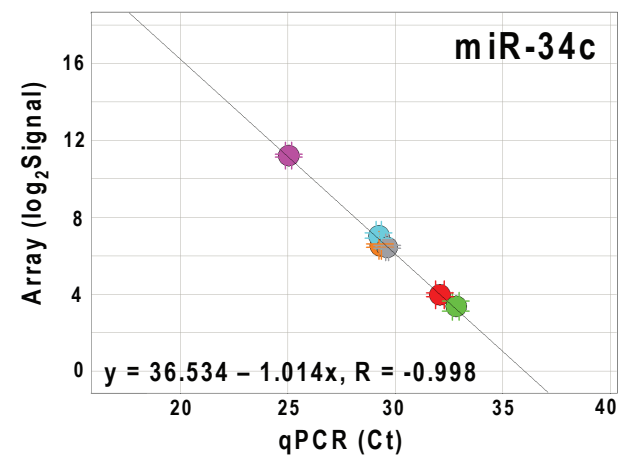

Supplement: Additional File 2 — Comparison of qPCR and microarray miRNA profiling for individual miRNAs. Scatter plots for 51 miRNAs not shown in Figure 1. [file 1472-6750-8-69-S2.pdf]

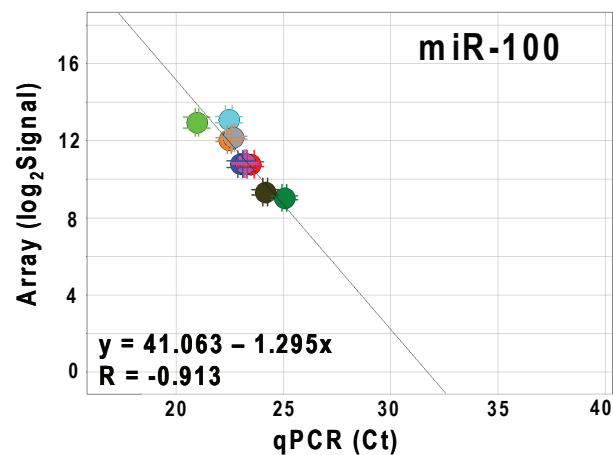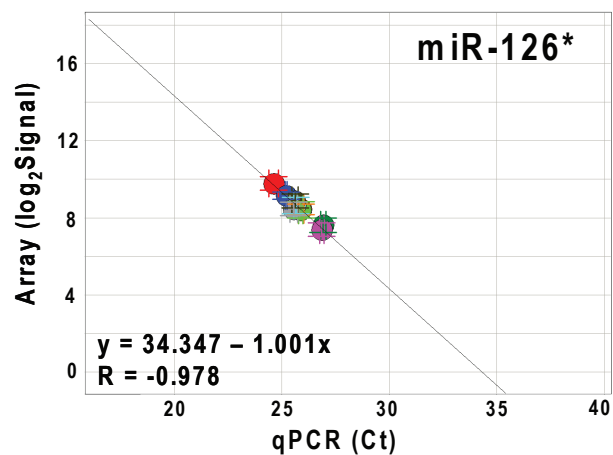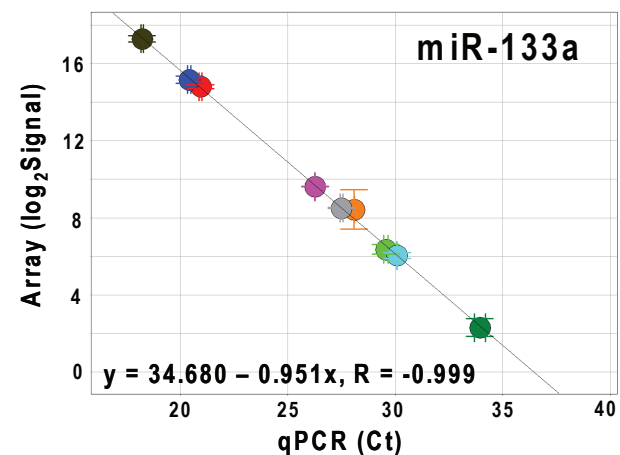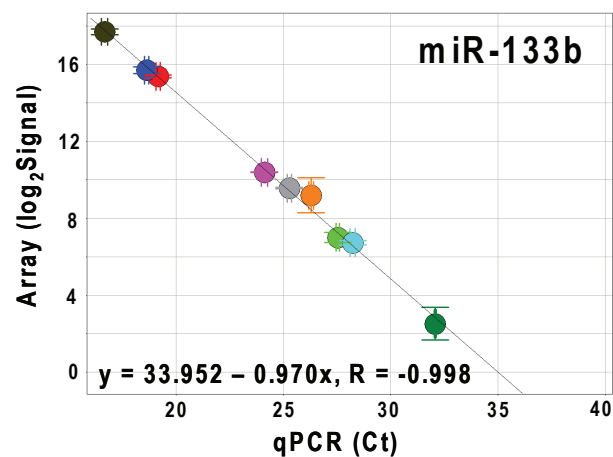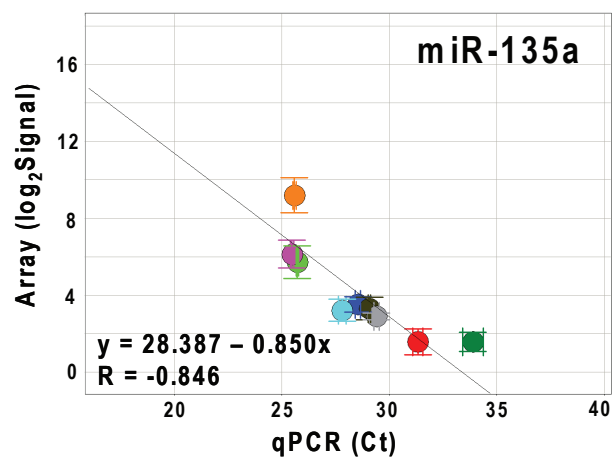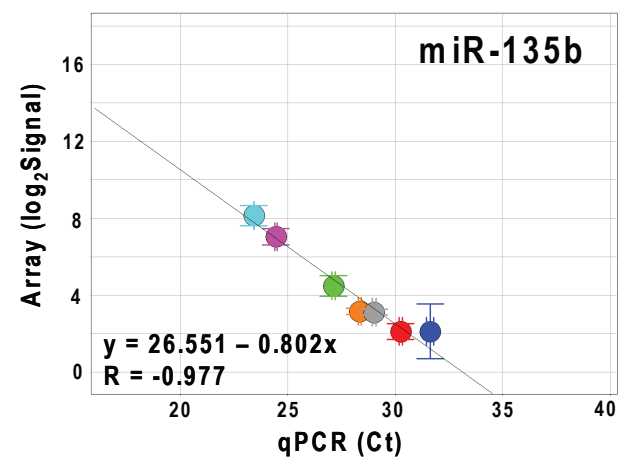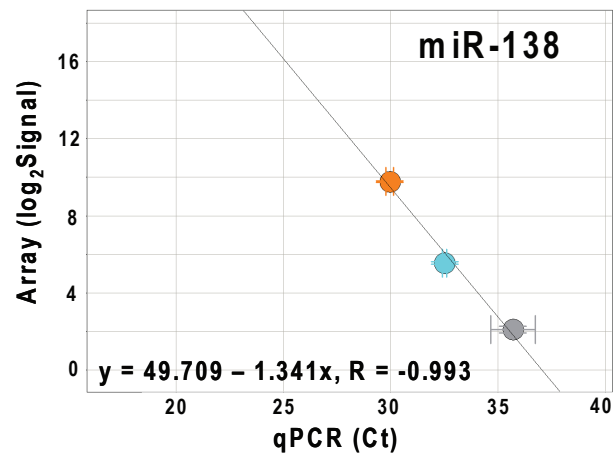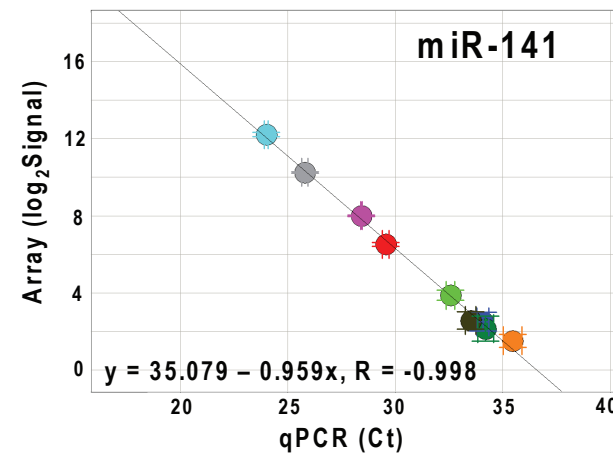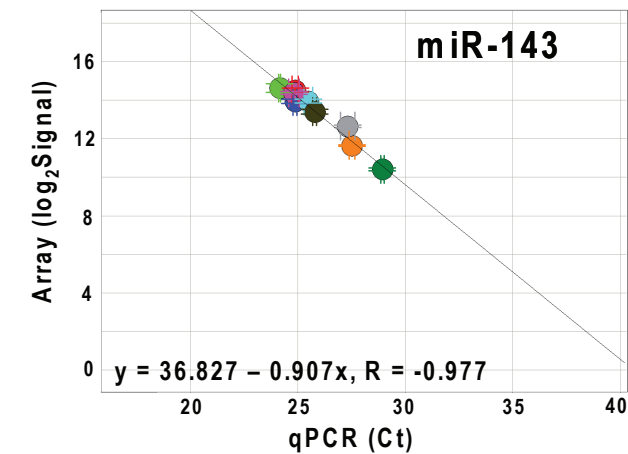

Supplement: Additional File 3 — Comparison of qPCR and microarray miRNA profiling for individual miRNAs. Scatter plots for 51 miRNAs not shown in Figure 1. [file 1472-6750-8-69-S3.pdf]

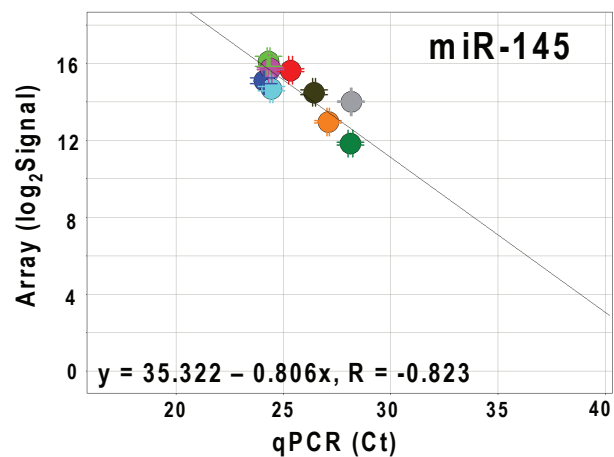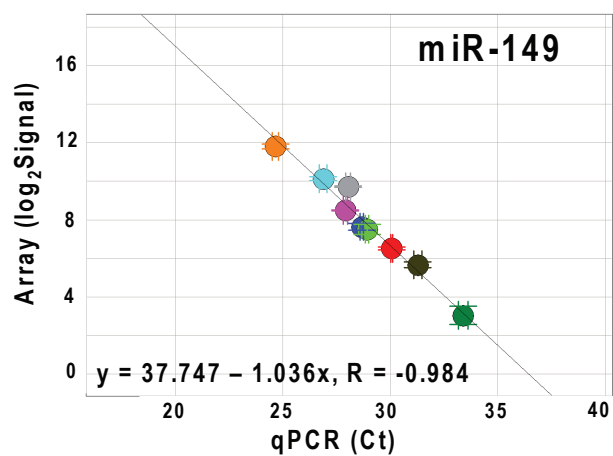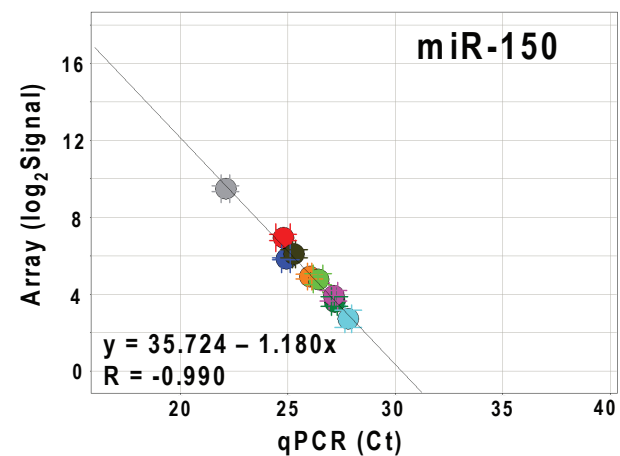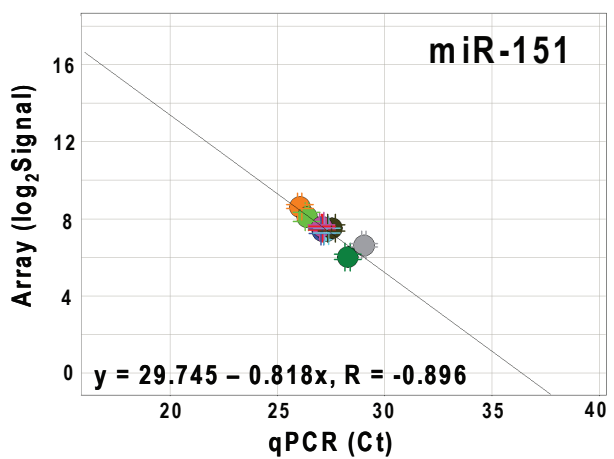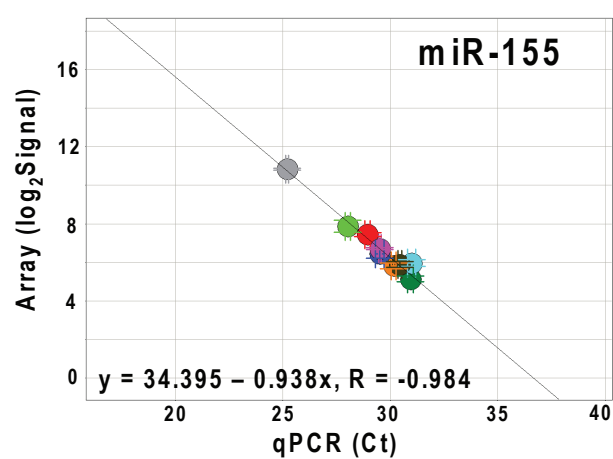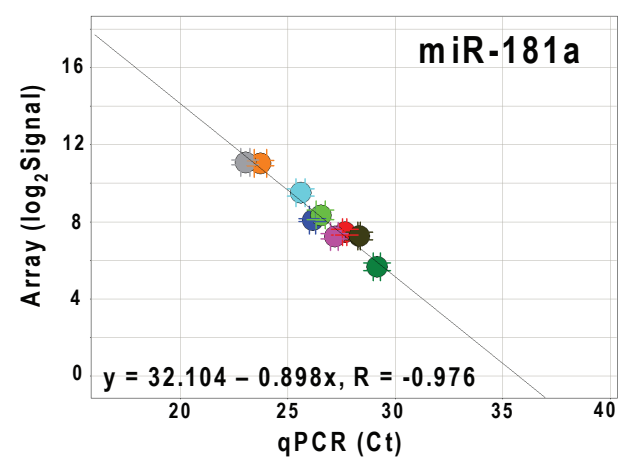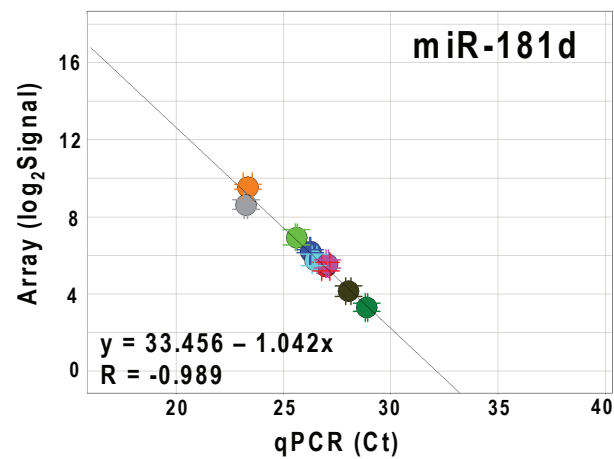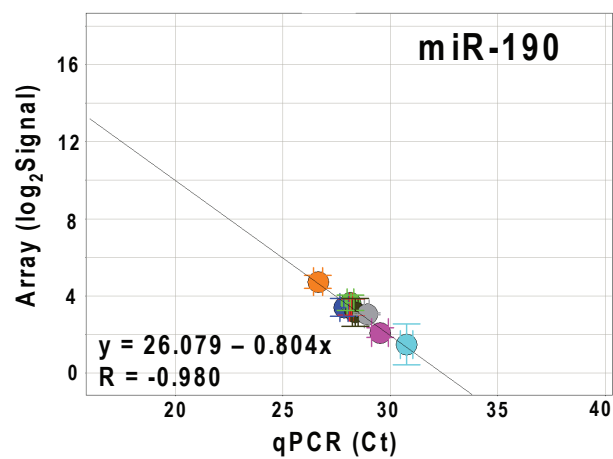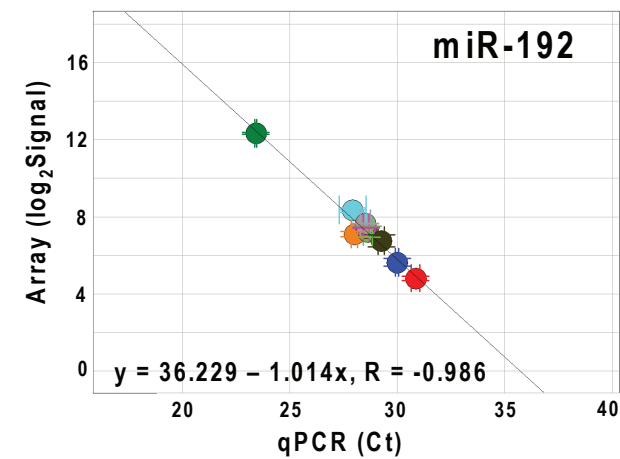

Supplement: Additional File 4 — Comparison of qPCR and microarray miRNA profiling for individual miRNAs. Scatter plots for 51 miRNAs not shown in Figure 1. [file 1472-6750-8-69-S4.pdf]

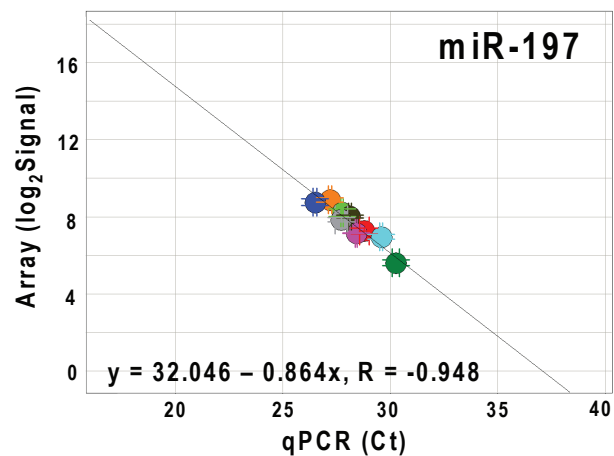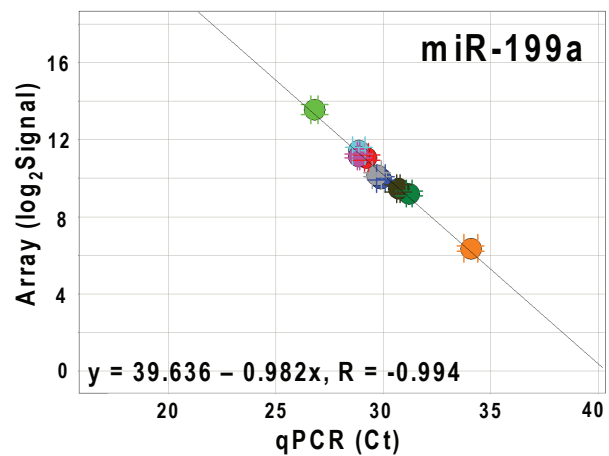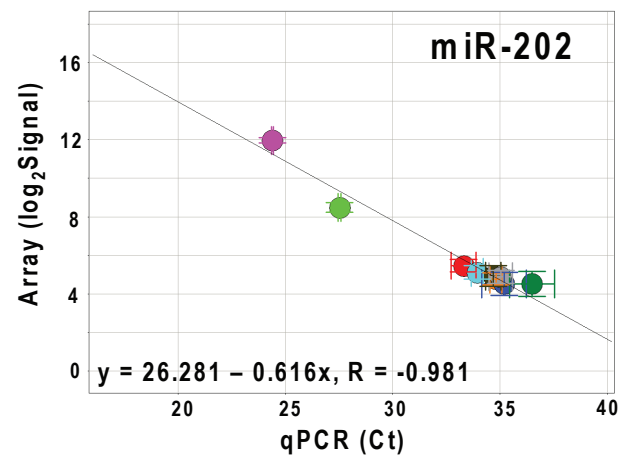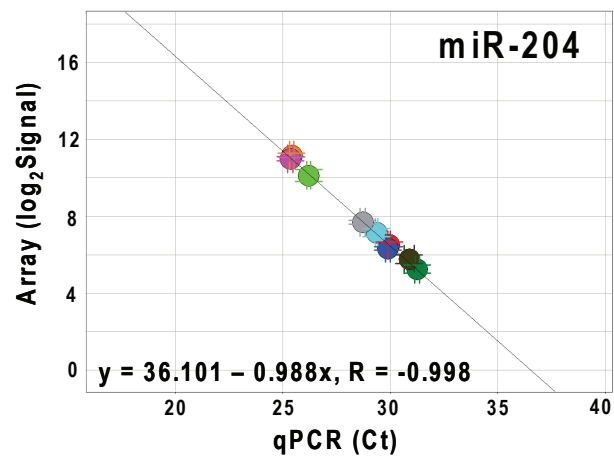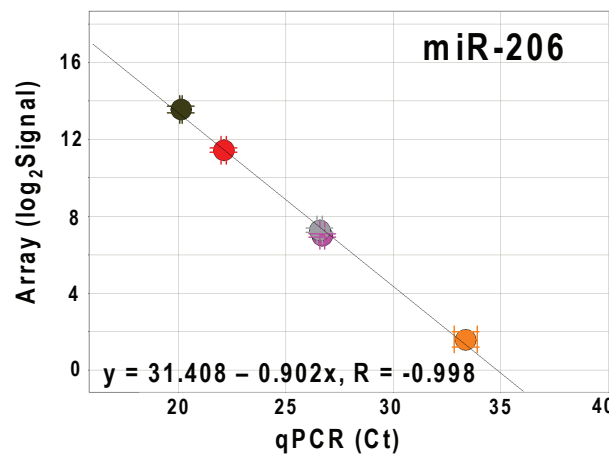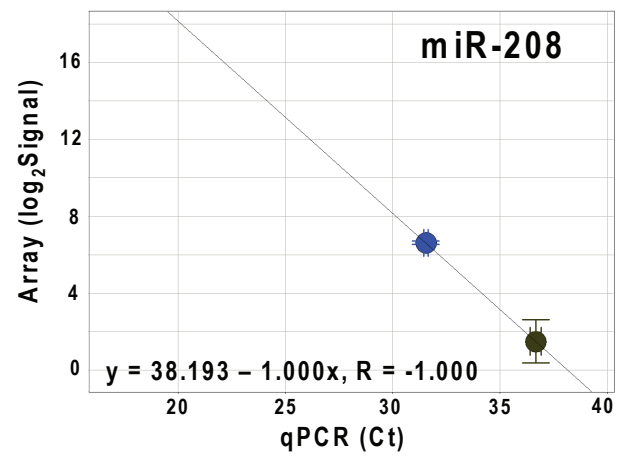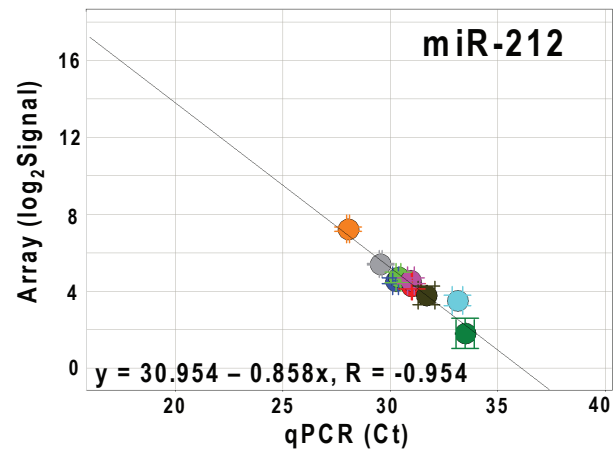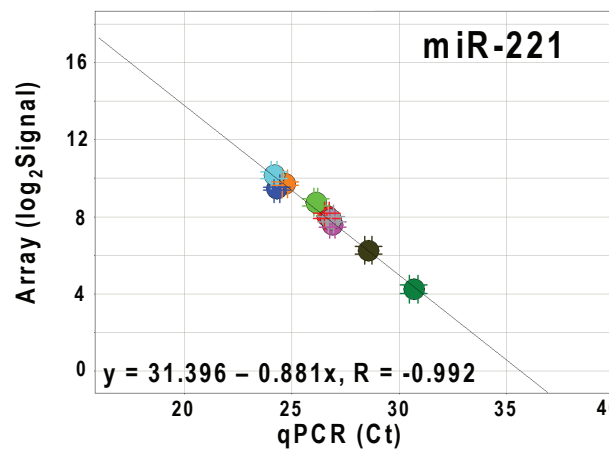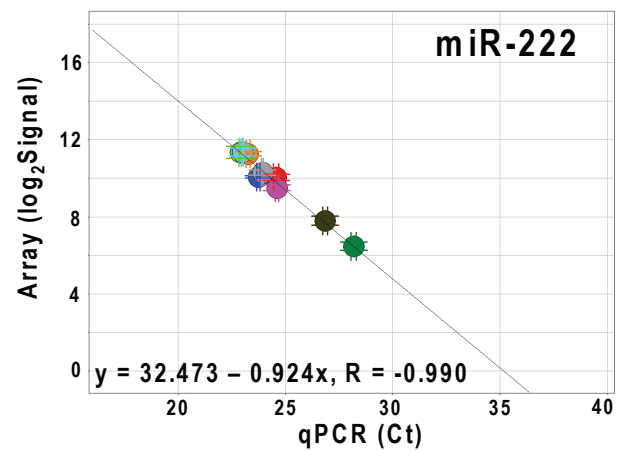

Supplement: Additional File 5 — Comparison of qPCR and microarray miRNA profiling for individual miRNAs. Scatter plots for 51 miRNAs not shown in Figure 1. [file 1472-6750-8-69-S5.pdf]

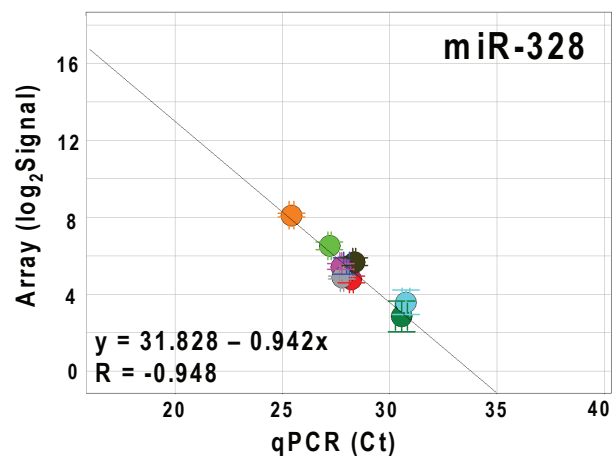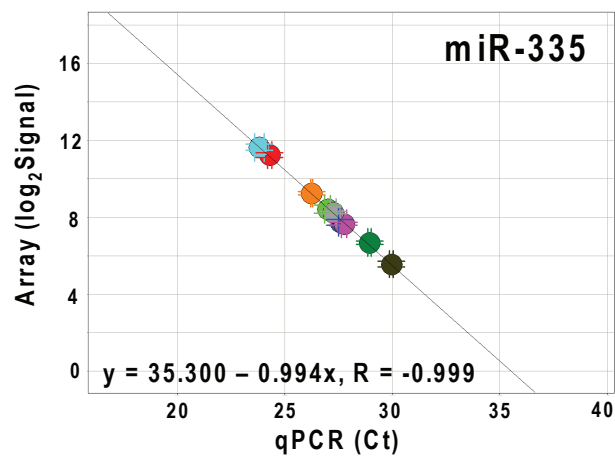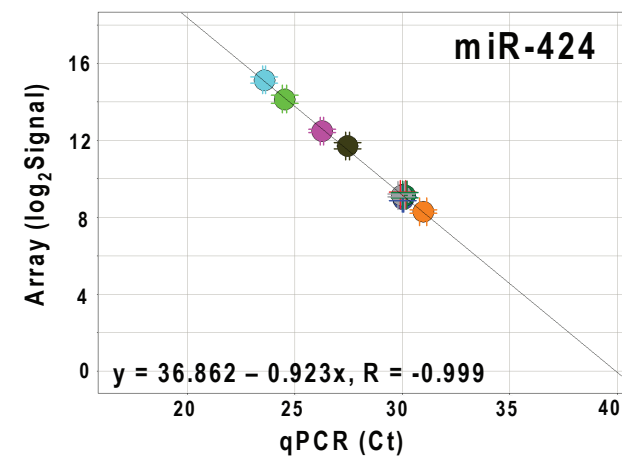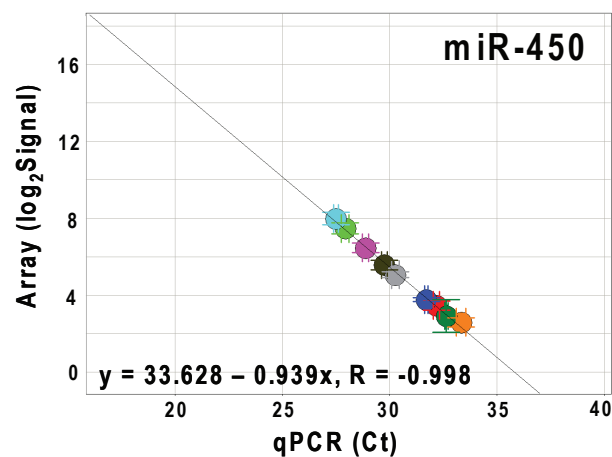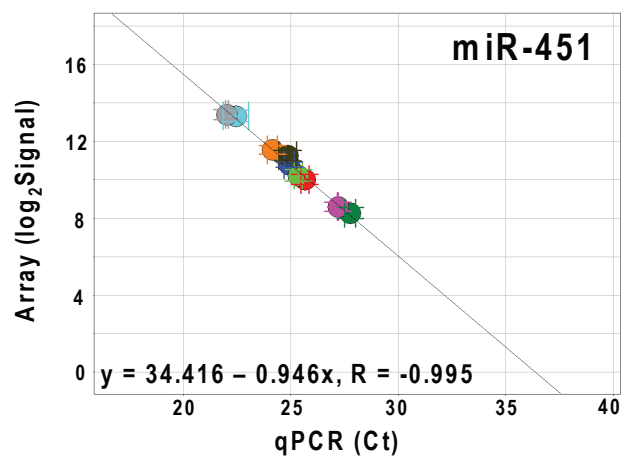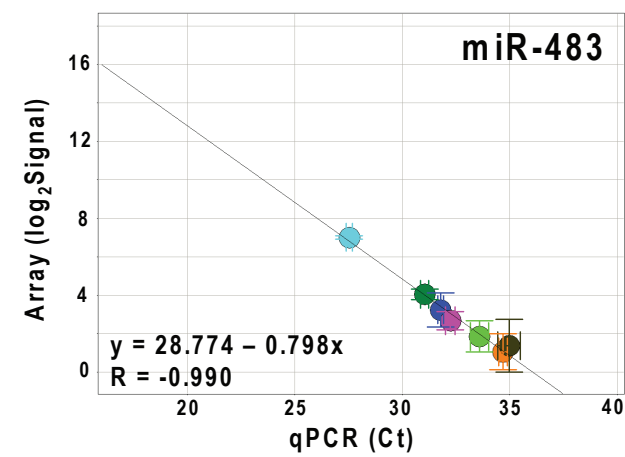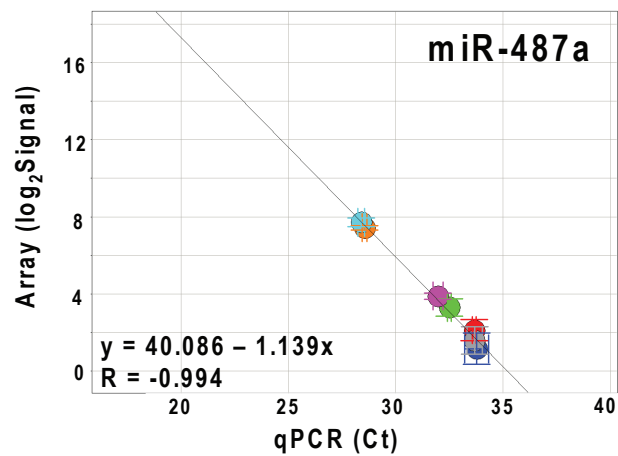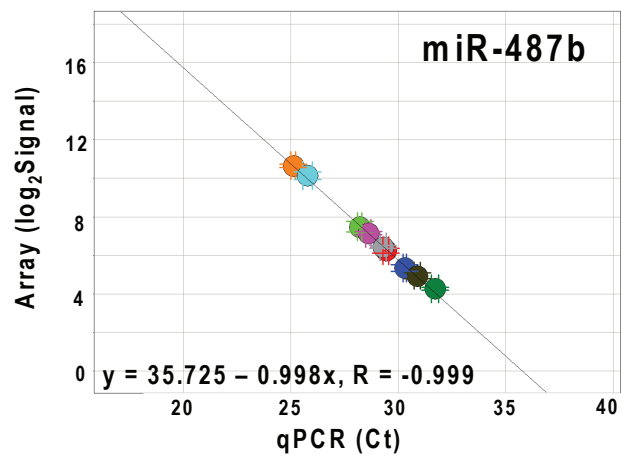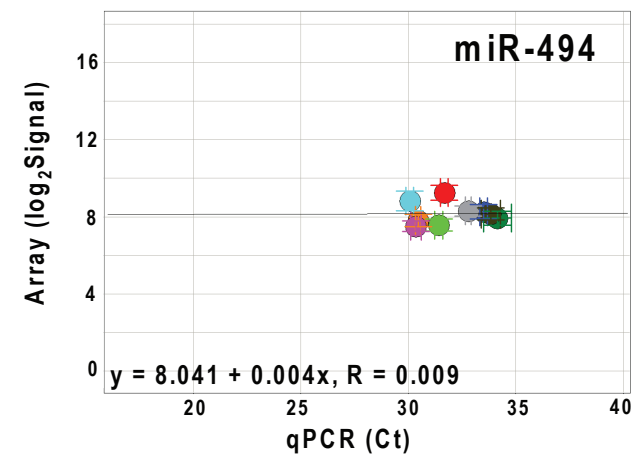

Supplement: Additional File 6 — Comparison of qPCR and microarray miRNA profiling for individual miRNAs. Scatter plots for 51 miRNAs not shown in Figure 1. [file 1472-6750-8-69-S6.pdf]

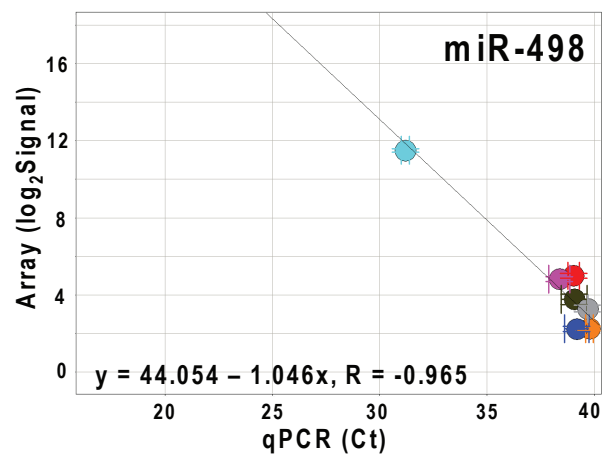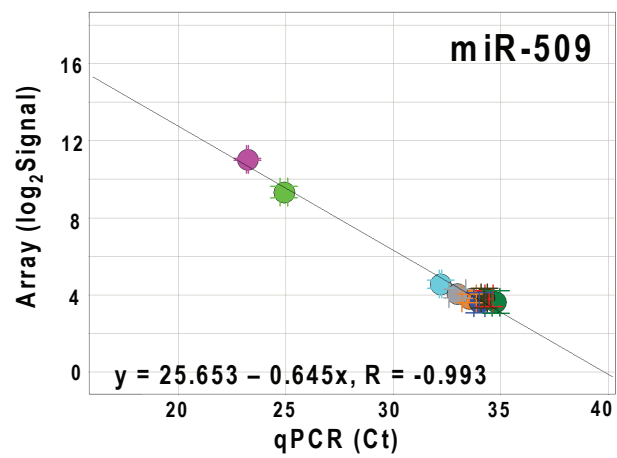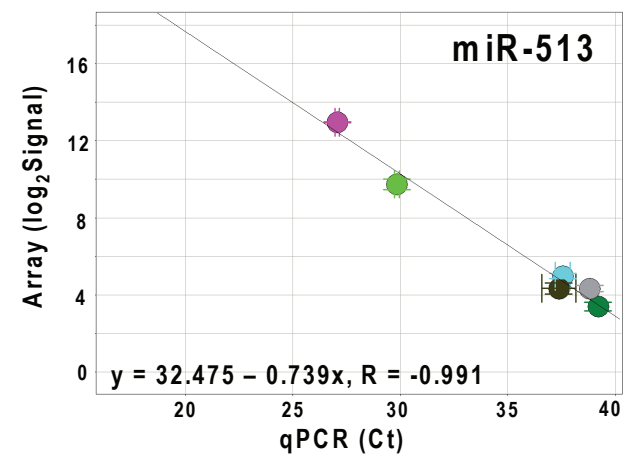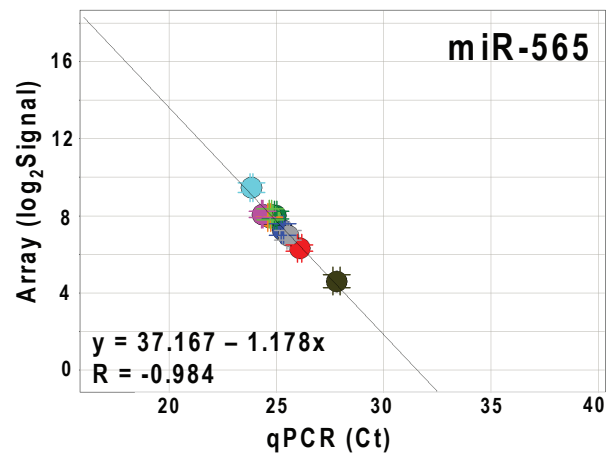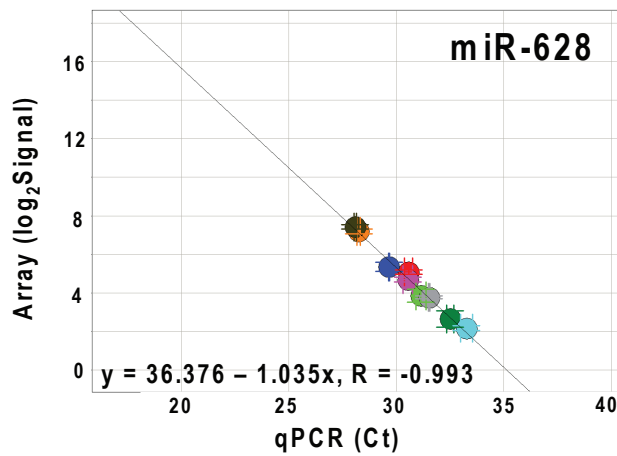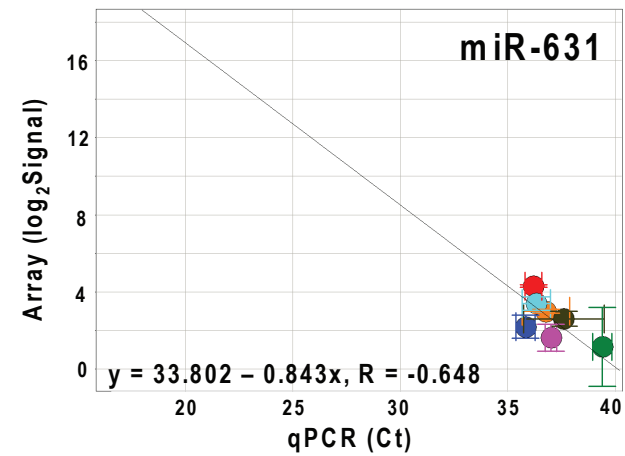

Supplement: Additional File 7 — Comparison of qPCR and microarray miRNA profiling for individual miRNAs. Scatter plots for 51 miRNAs not shown in Figure 1. [file 1472-6750-8-69-S7.pdf]

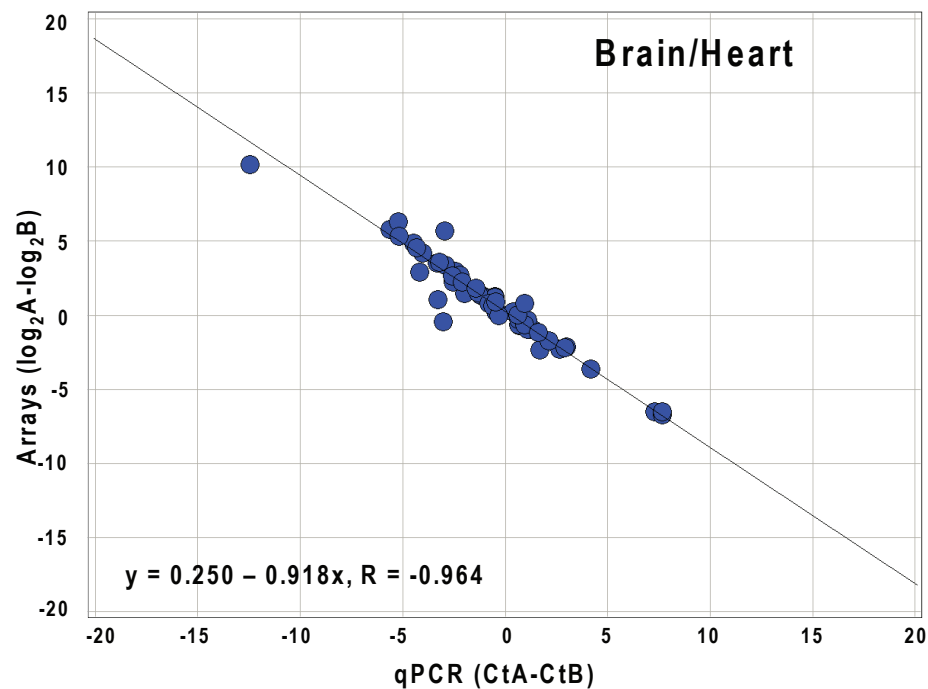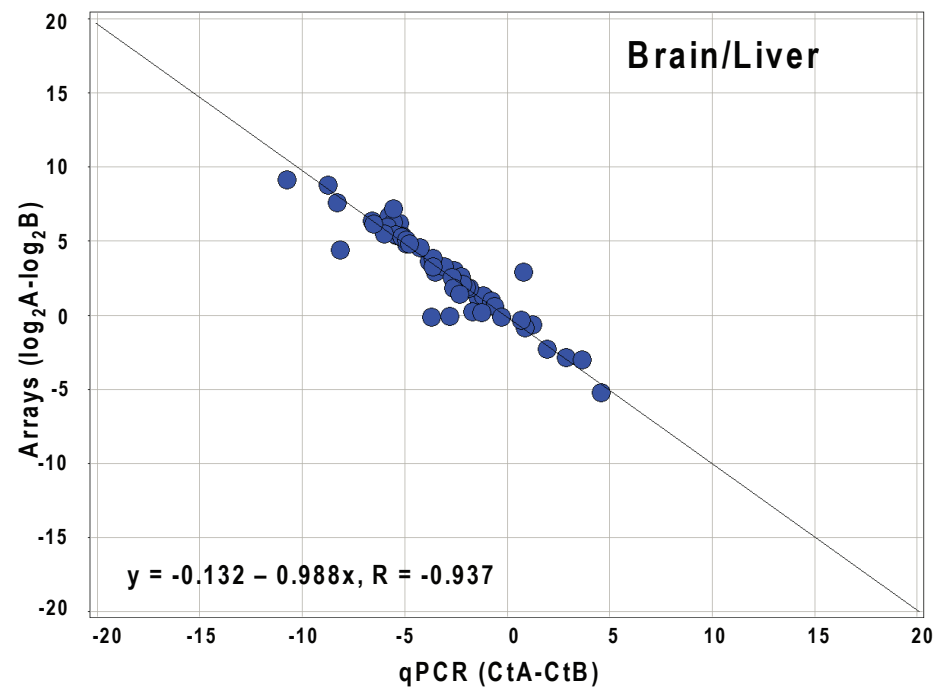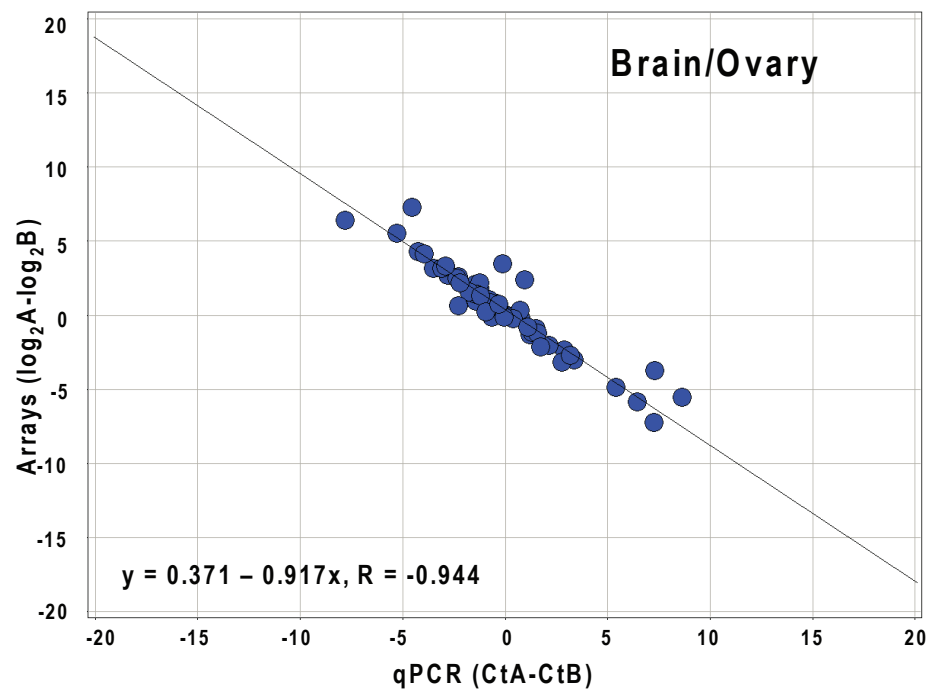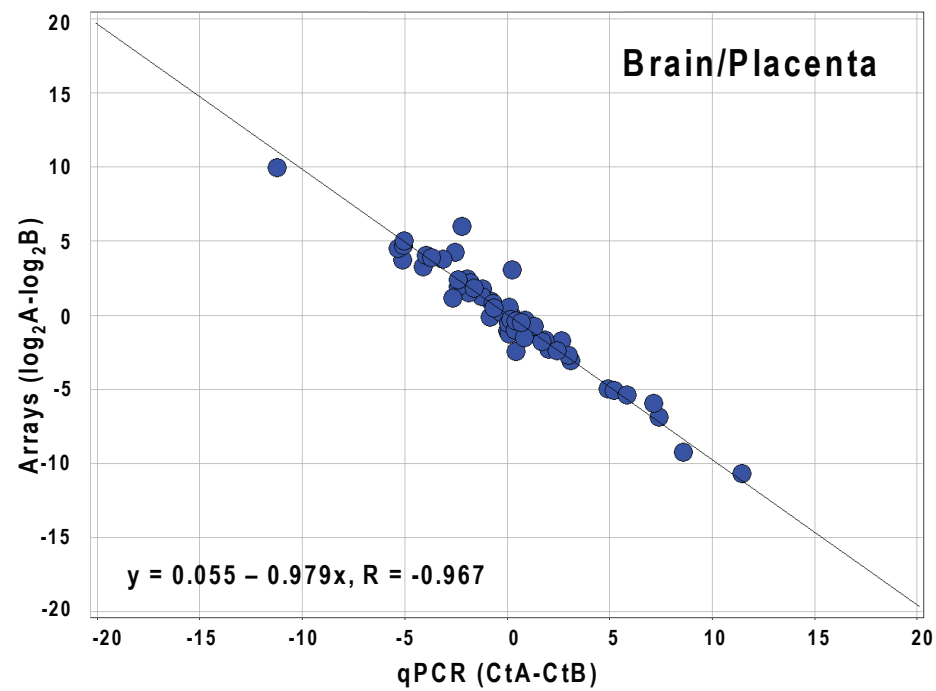

Supplement: Additional File 8 — Comparison of qPCR and microarray miRNA profiling for 60 miRNAs in tissue pairs. Scatter plots for 32 tissue pairs not shown in Figure 3. [file 1472-6750-8-69-S8.pdf]

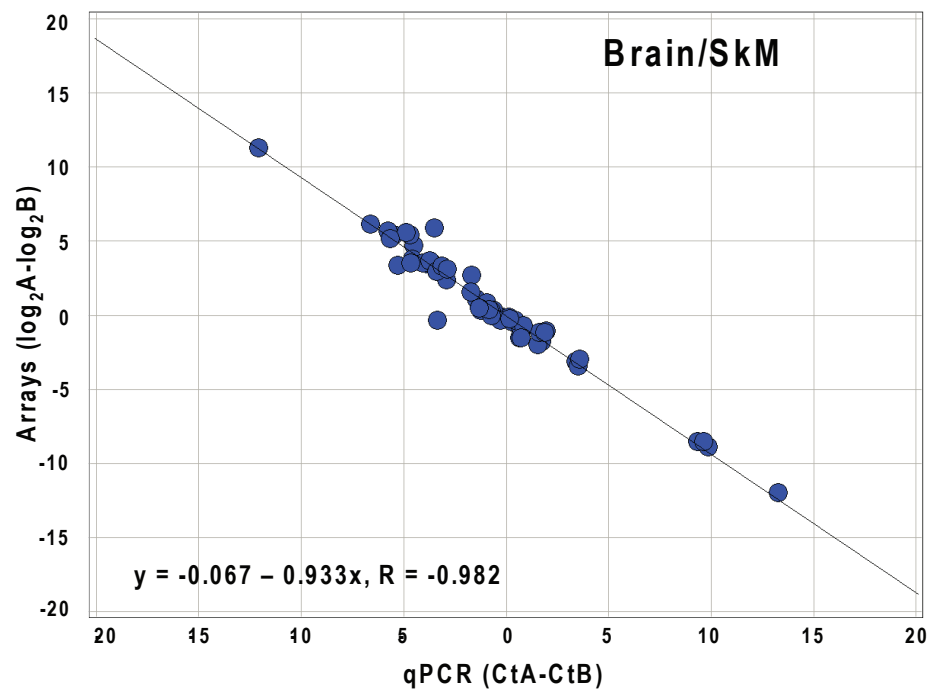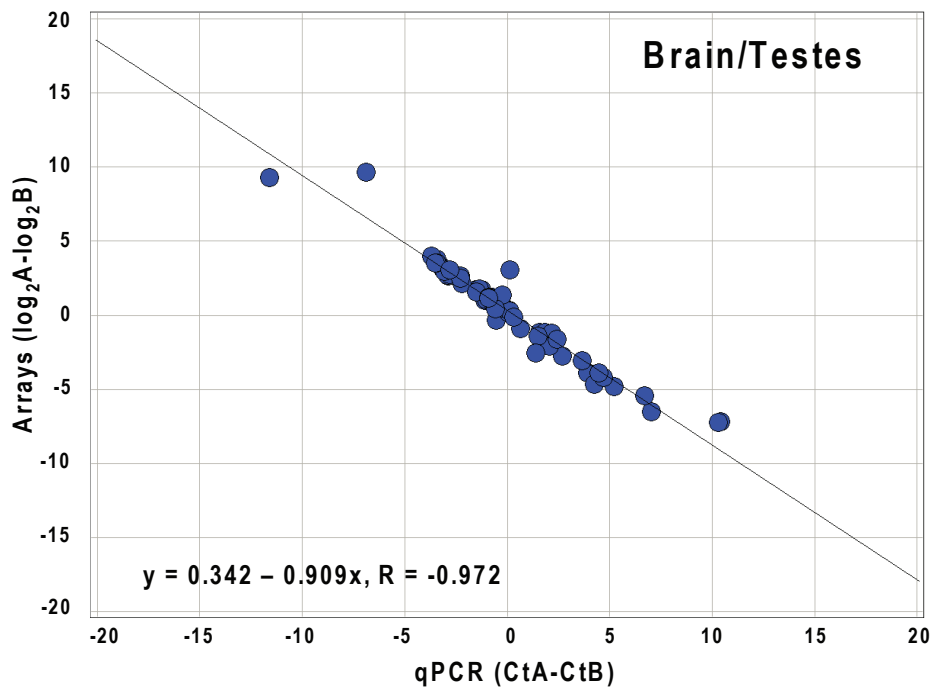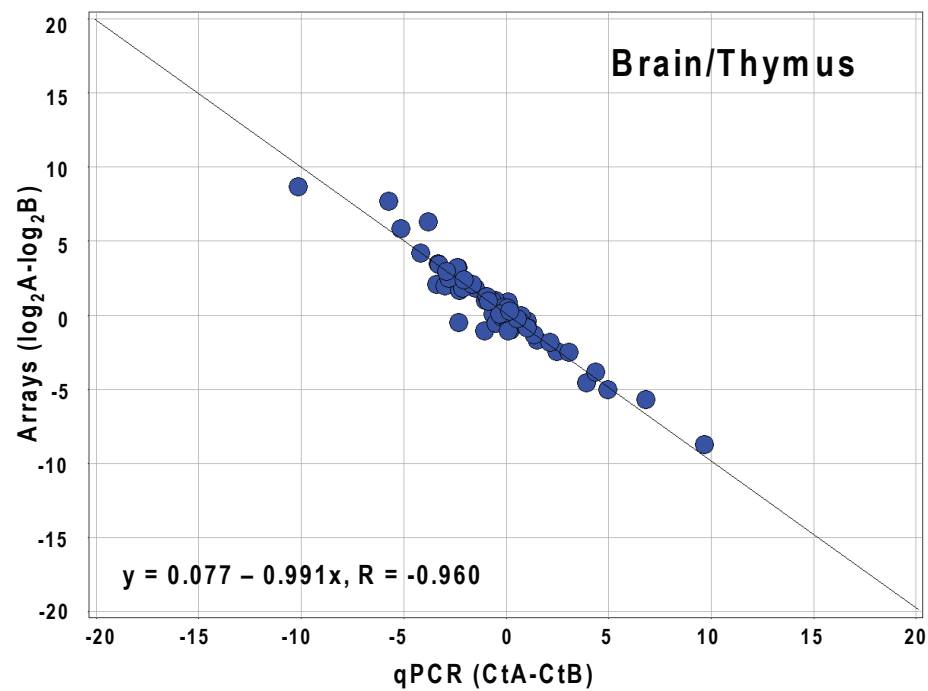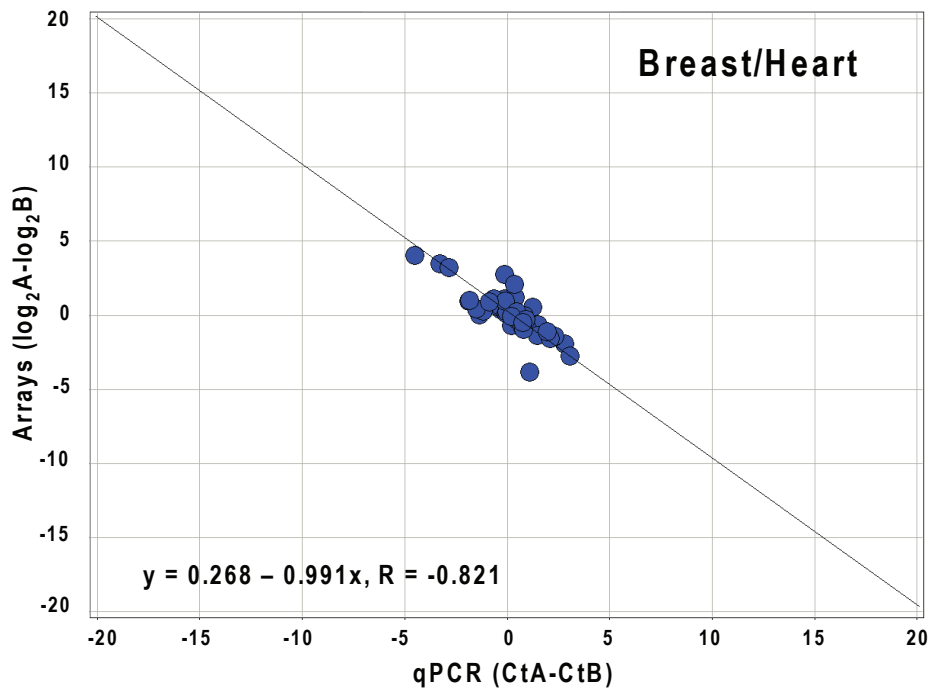

Supplement: Additional File 9 — Comparison of qPCR and microarray miRNA profiling for 60 miRNAs in tissue pairs. Scatter plots for 32 tissue pairs not shown in Figure 3. [file 1472-6750-8-69-S9.pdf]

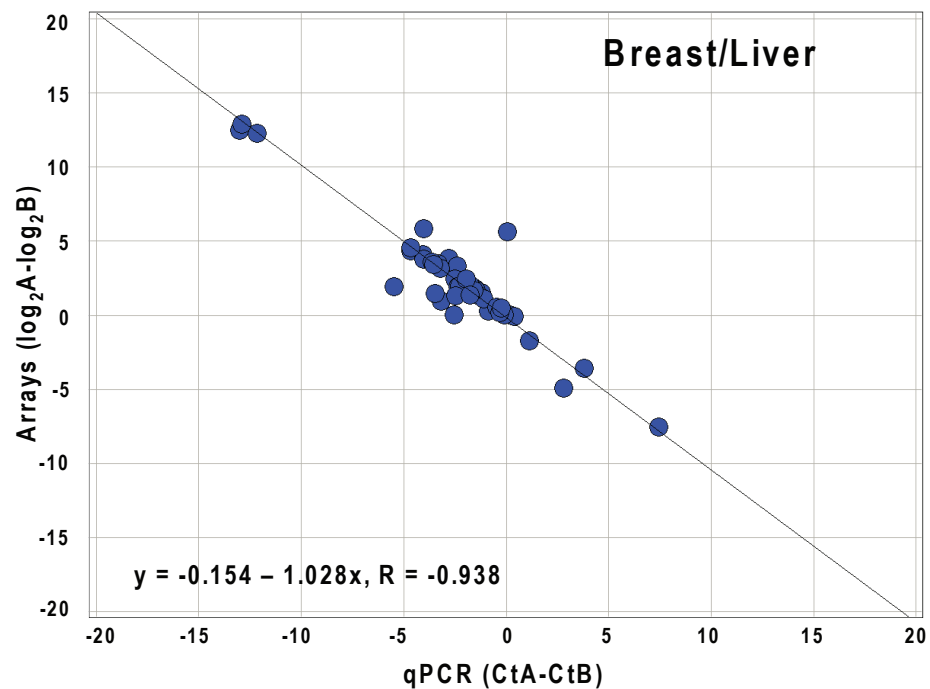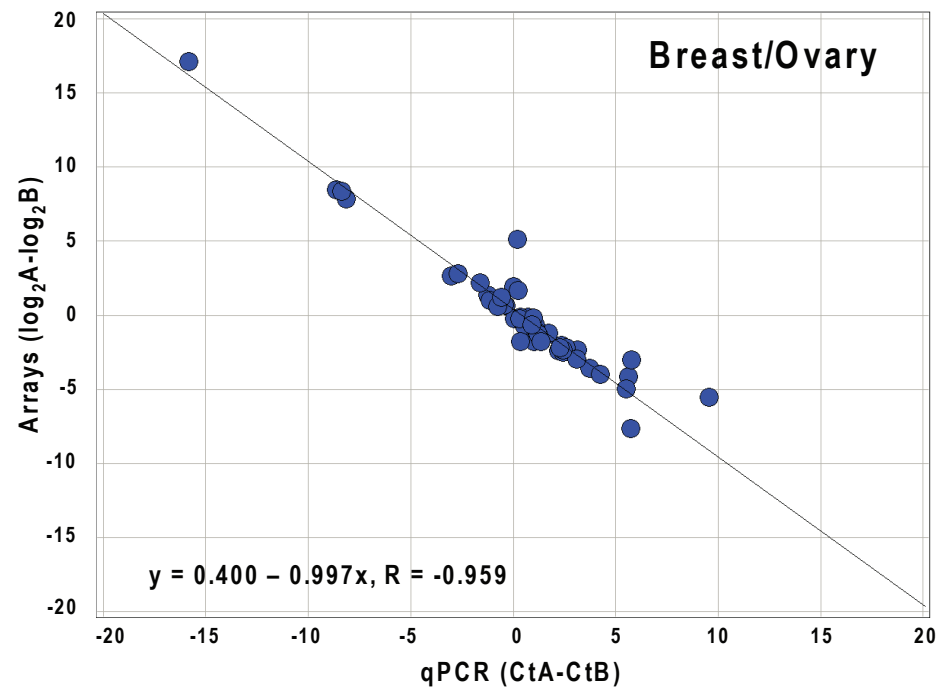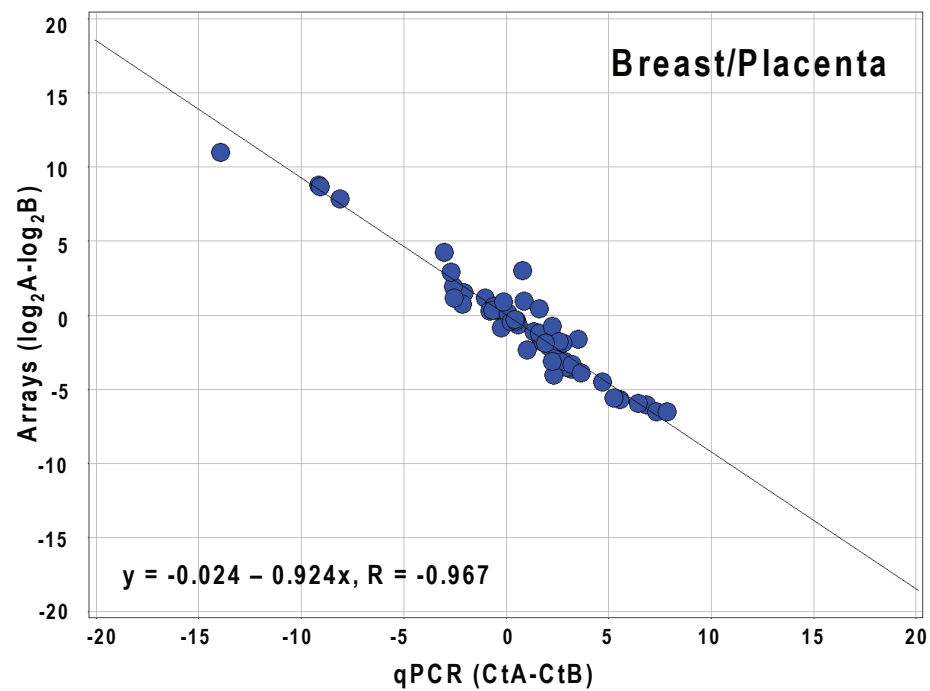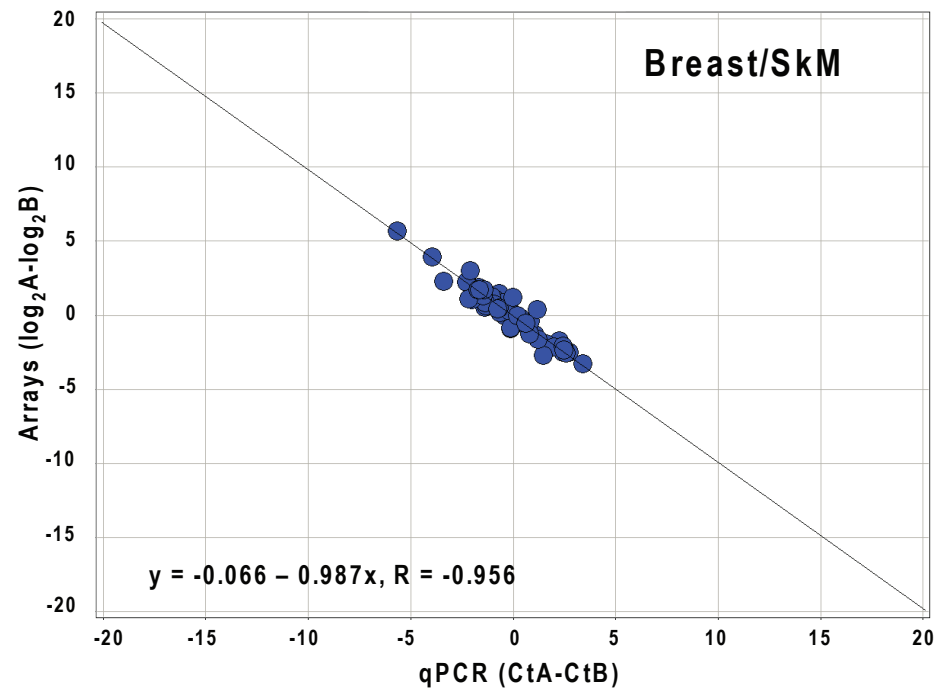

Supplement: Additional File 10 — Comparison of qPCR and microarray miRNA profiling for 60 miRNAs in tissue pairs. Scatter plots for 32 tissue pairs not shown in Figure 3. [file 1472-6750-8-69-S10.pdf]

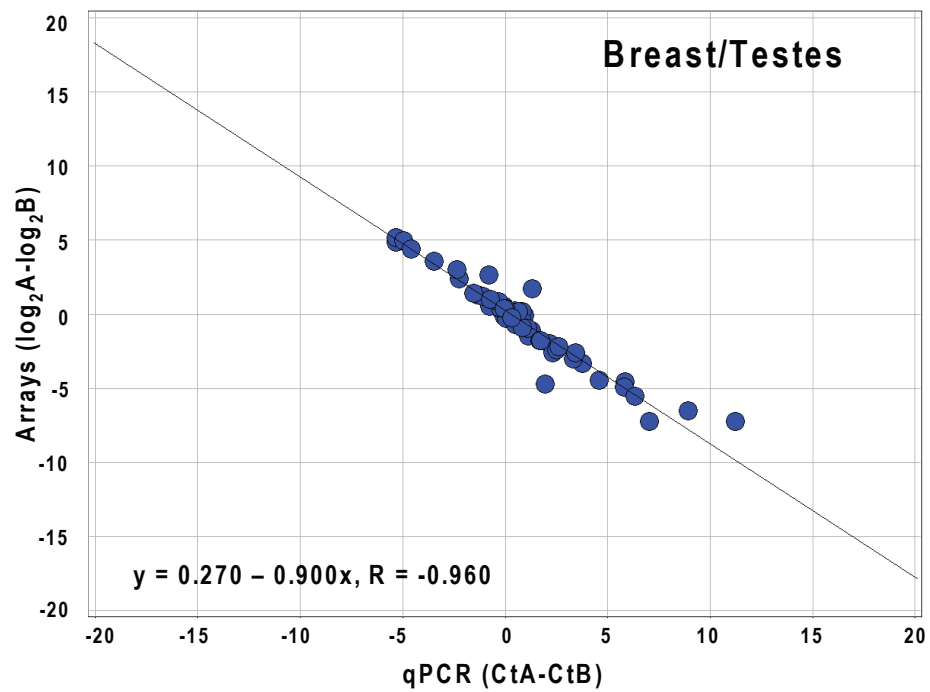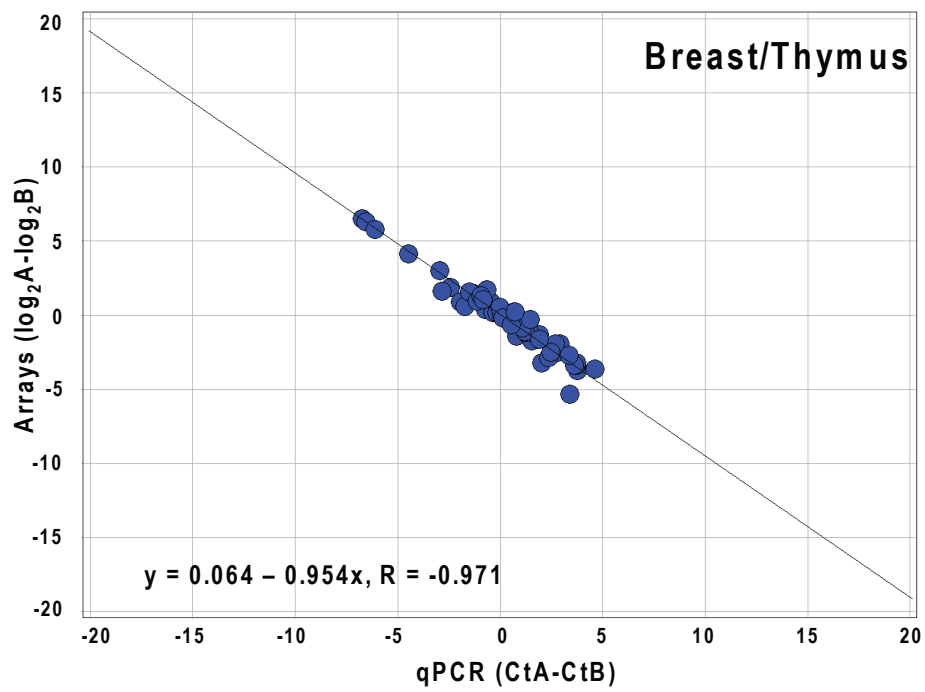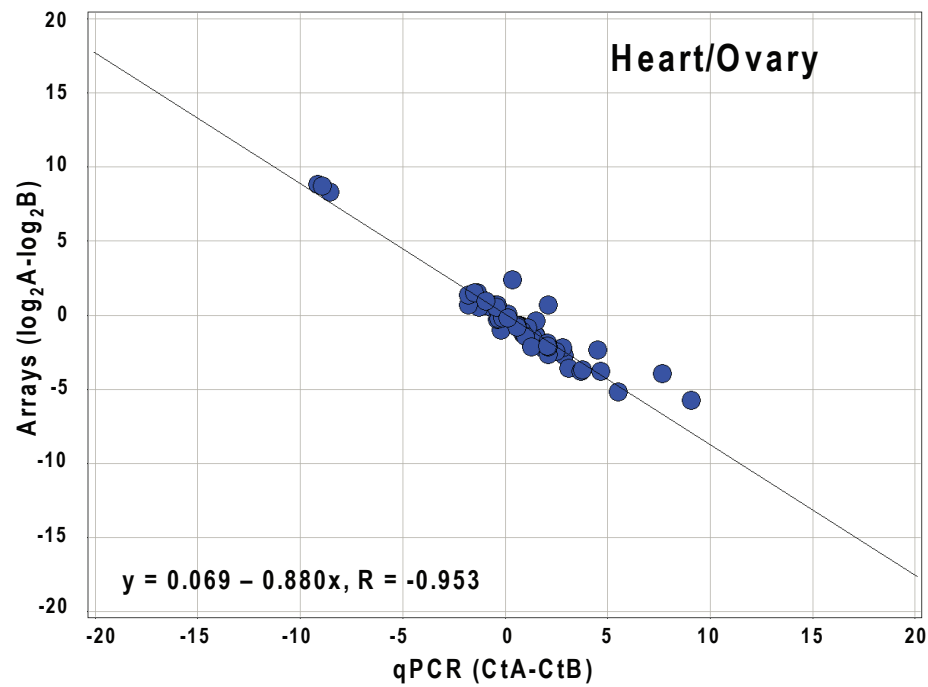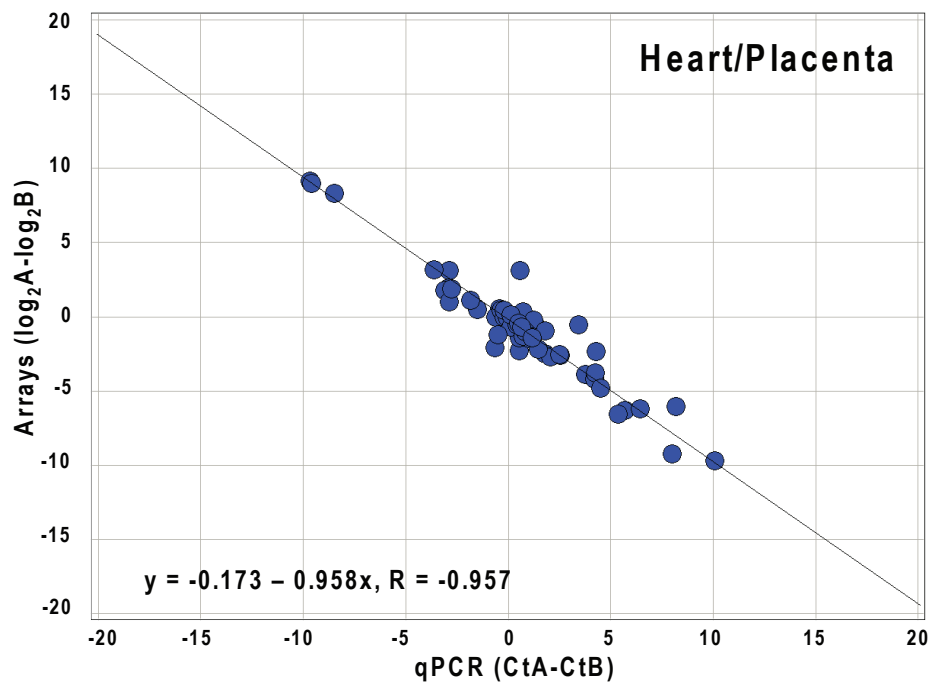

Supplement: Additional File 11 — Comparison of qPCR and microarray miRNA profiling for 60 miRNAs in tissue pairs. Scatter plots for 32 tissue pairs not shown in Figure 3. [file 1472-6750-8-69-S11.pdf]

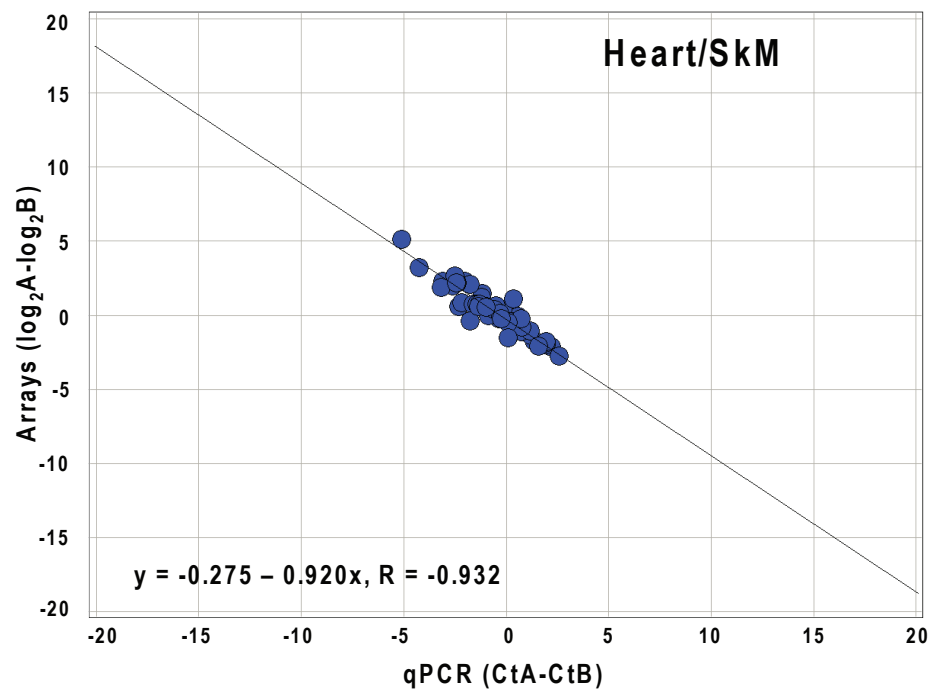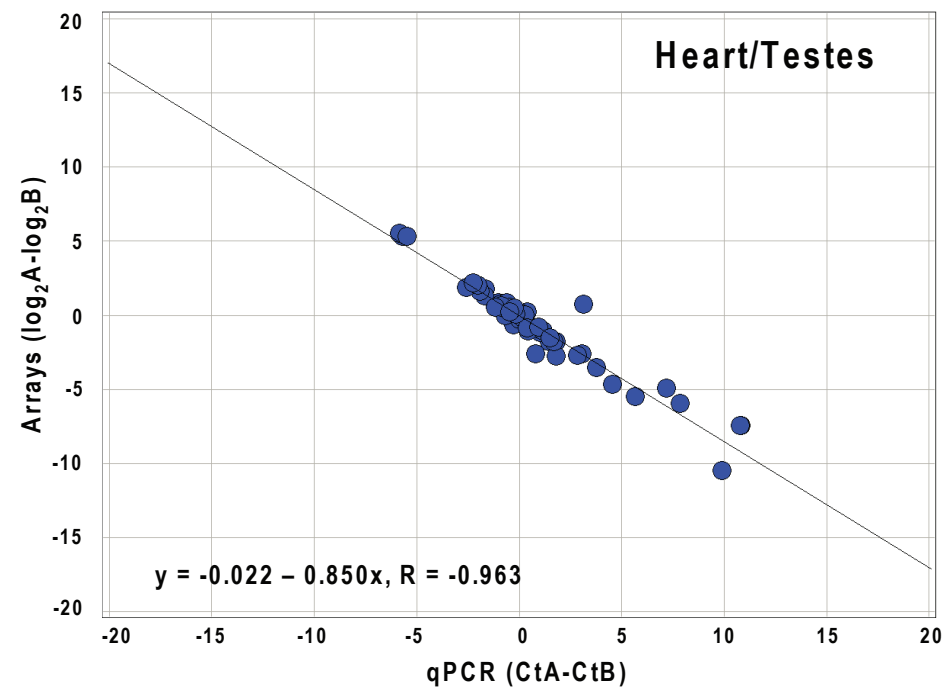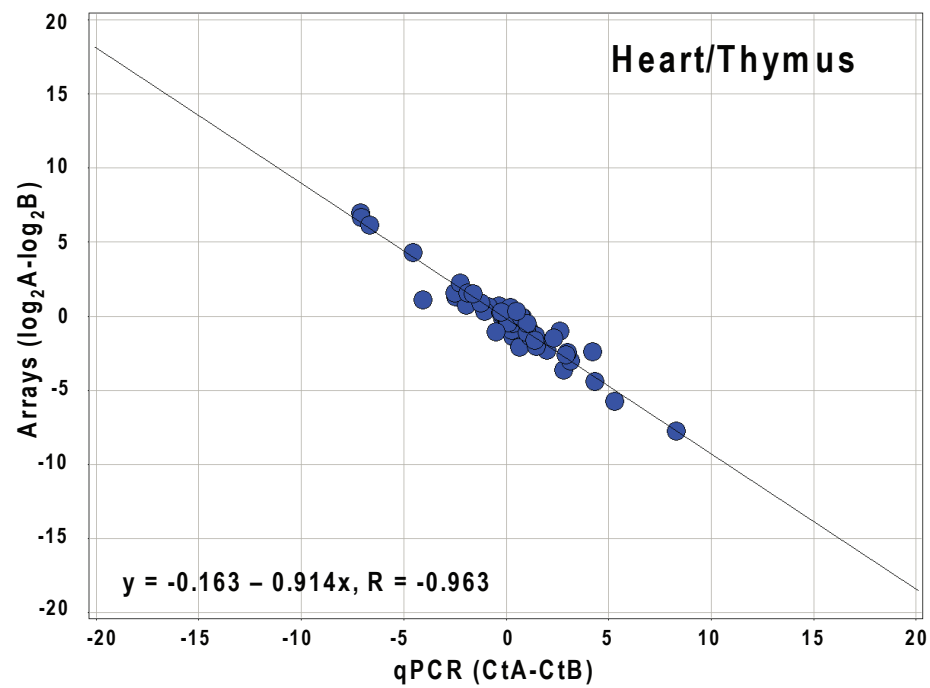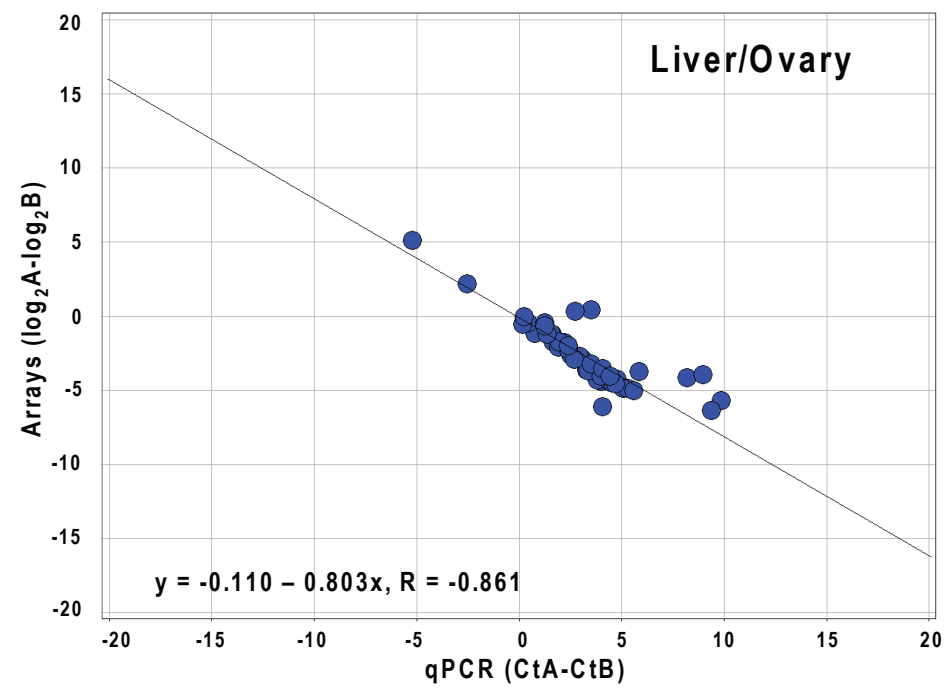

Supplement: Additional File 12 — Comparison of qPCR and microarray miRNA profiling for 60 miRNAs in tissue pairs. Scatter plots for 32 tissue pairs not shown in Figure 3. [file 1472-6750-8-69-S12.pdf]

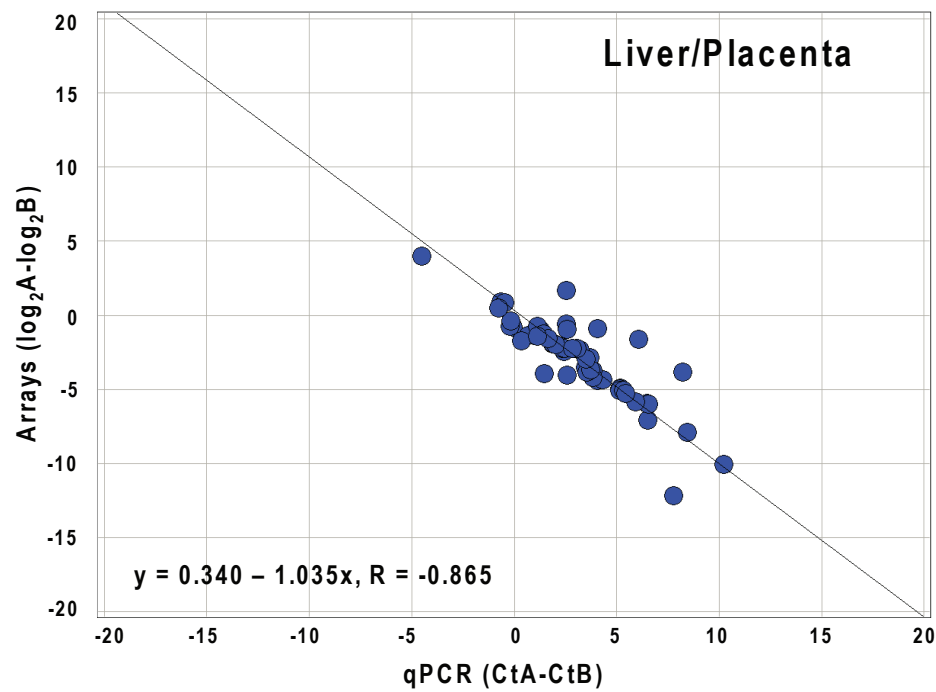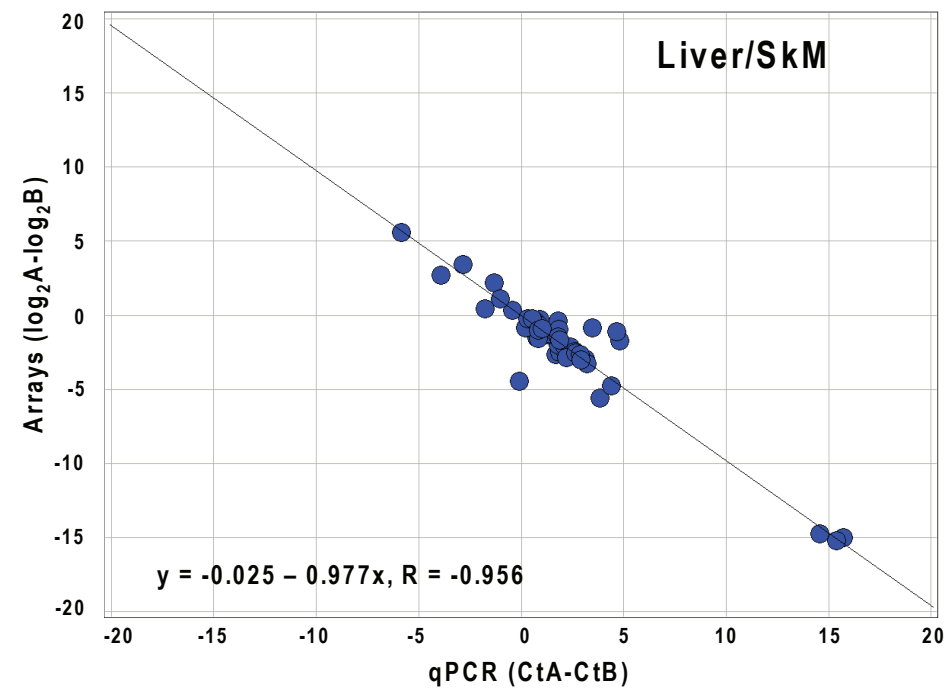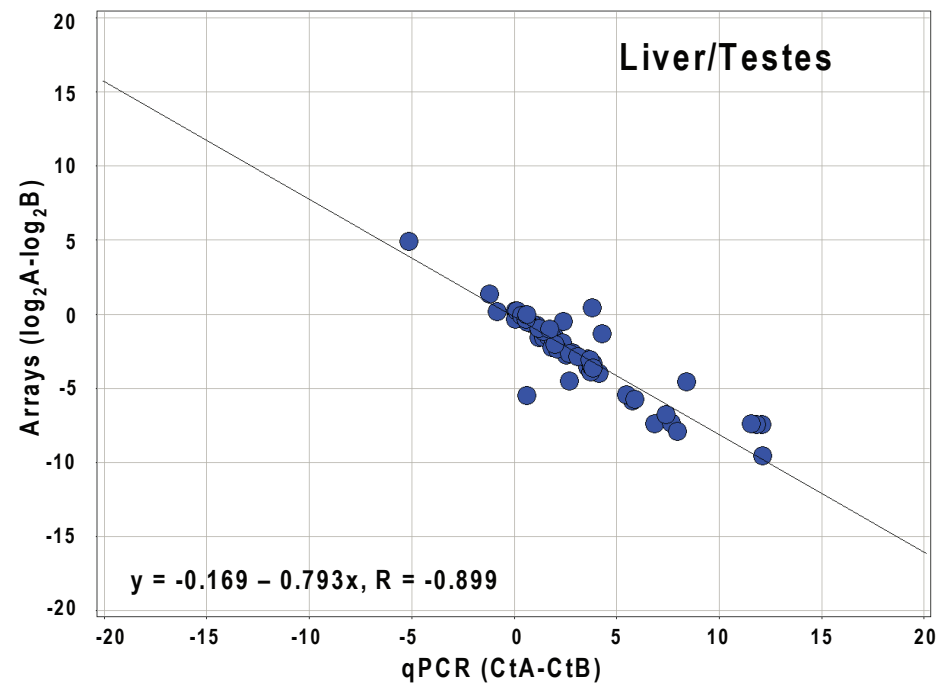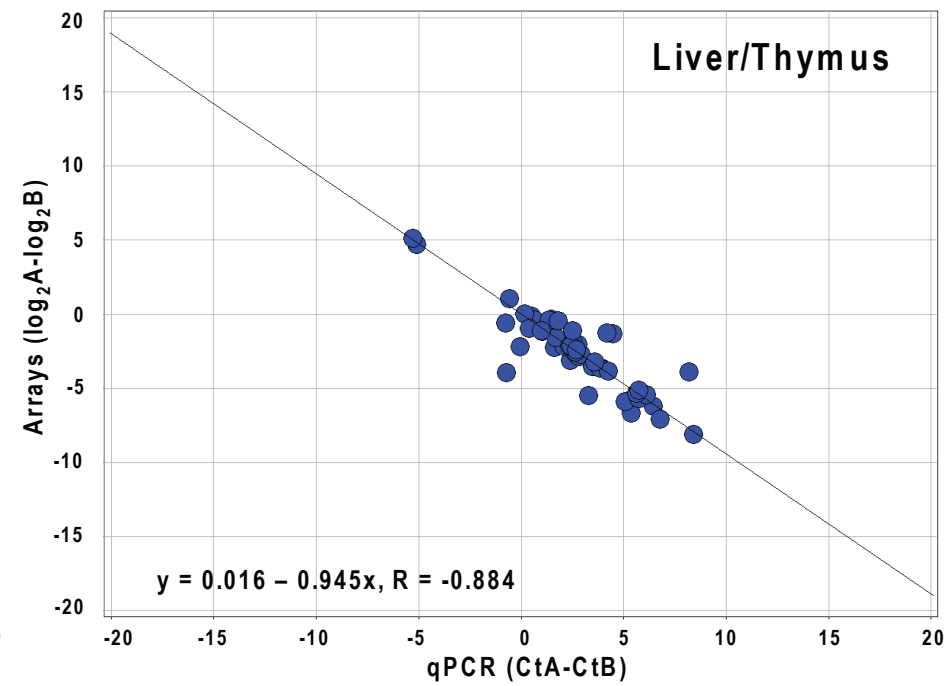

Supplement: Additional File 13 — Comparison of qPCR and microarray miRNA profiling for 60 miRNAs in tissue pairs. Scatter plots for 32 tissue pairs not shown in Figure 3. [file 1472-6750-8-69-S13.pdf]

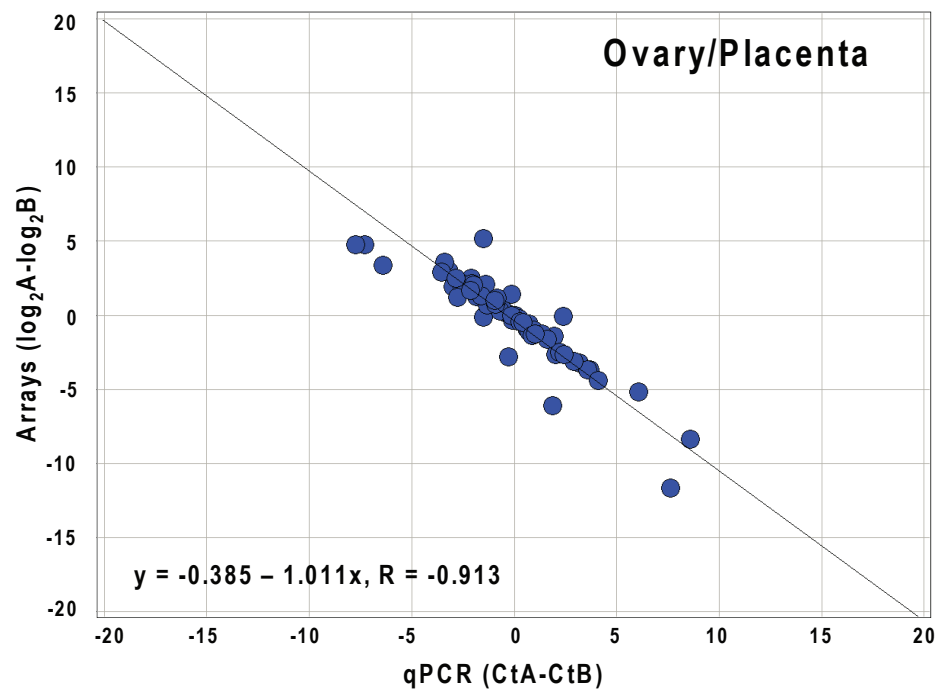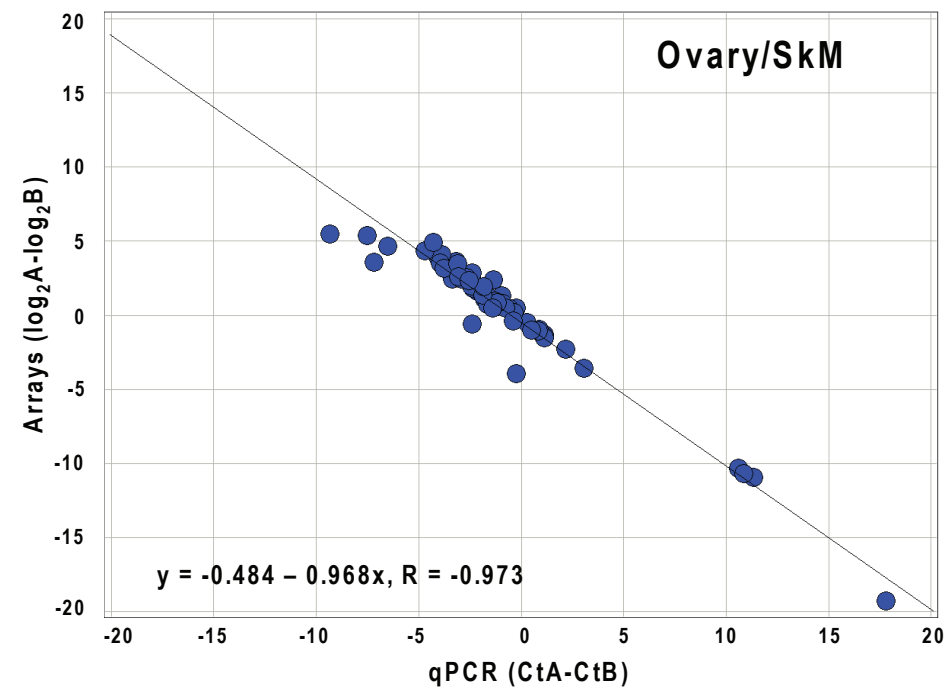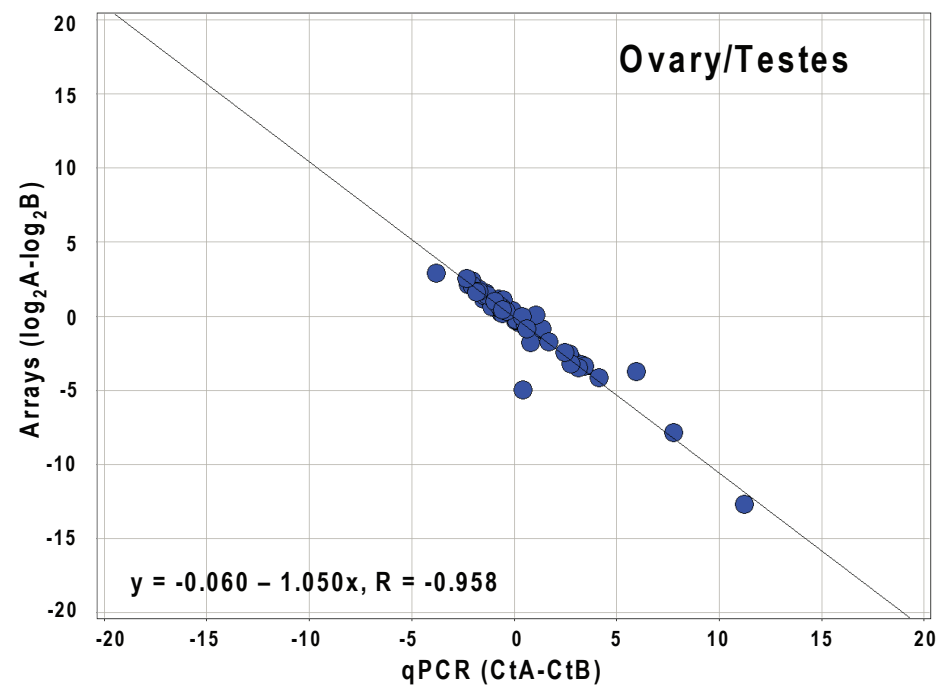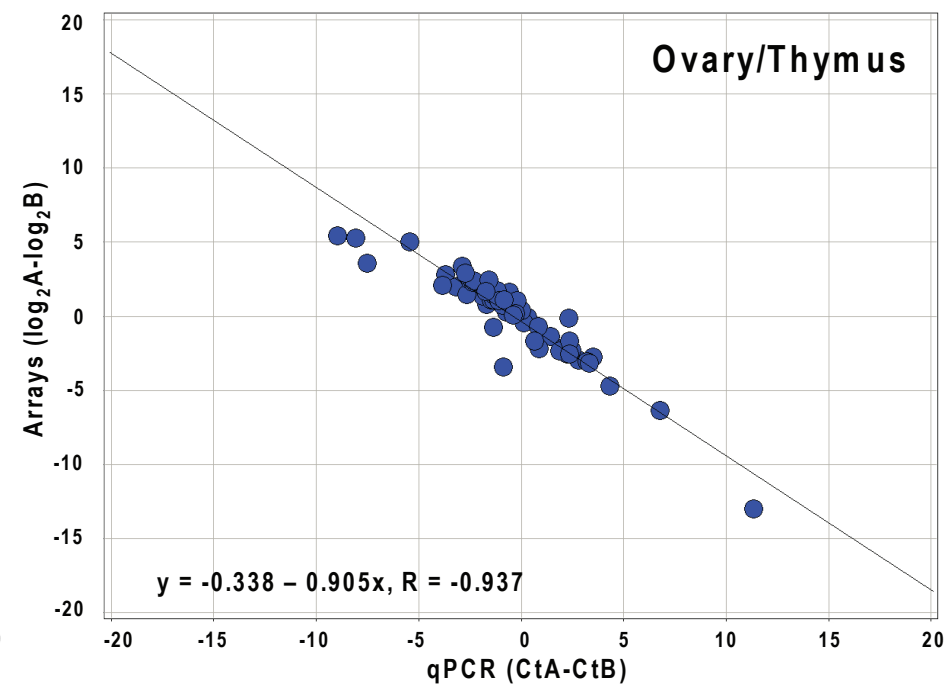

Supplement: Additional File 14 — Comparison of qPCR and microarray miRNA profiling for 60 miRNAs in tissue pairs. Scatter plots for 32 tissue pairs not shown in Figure 3. [file 1472-6750-8-69-S14.pdf]

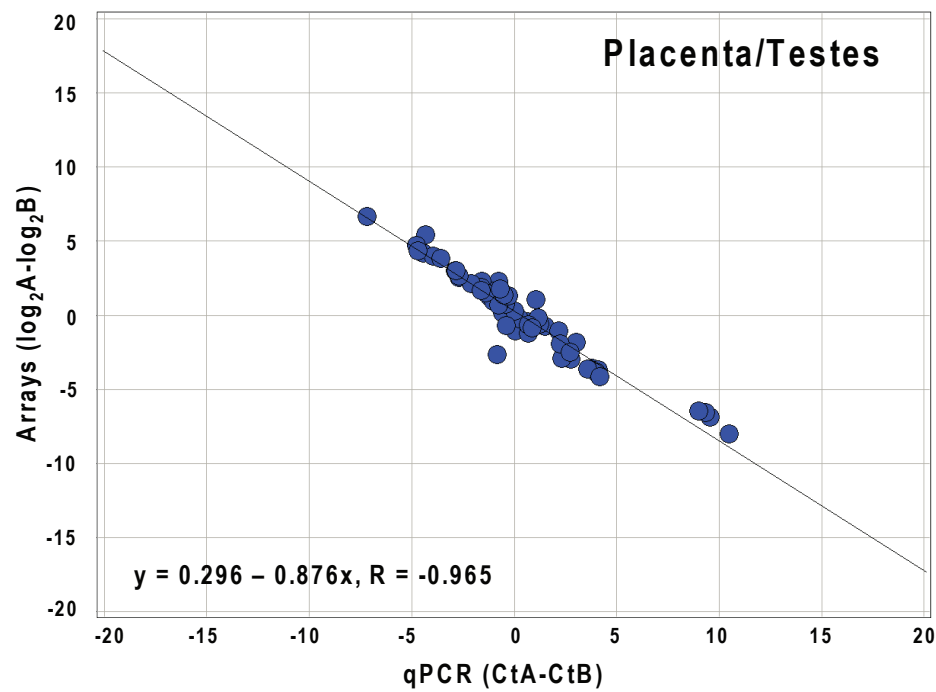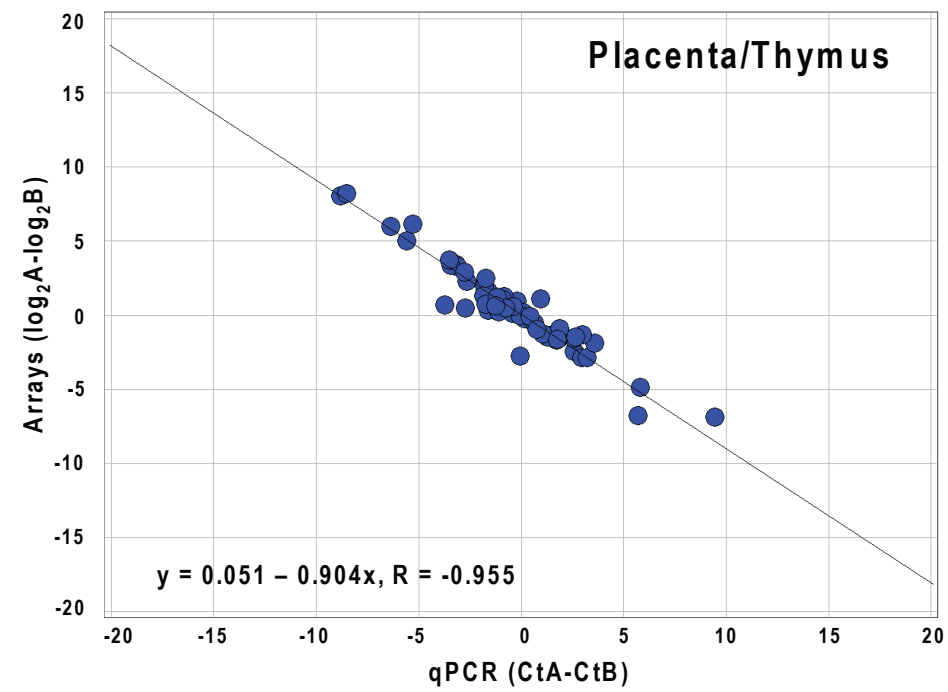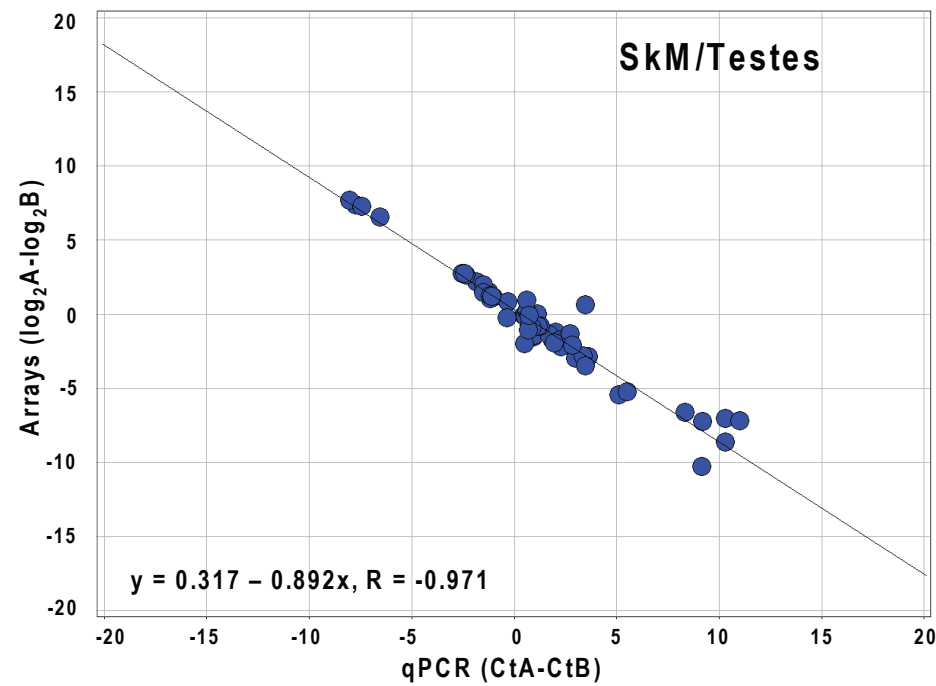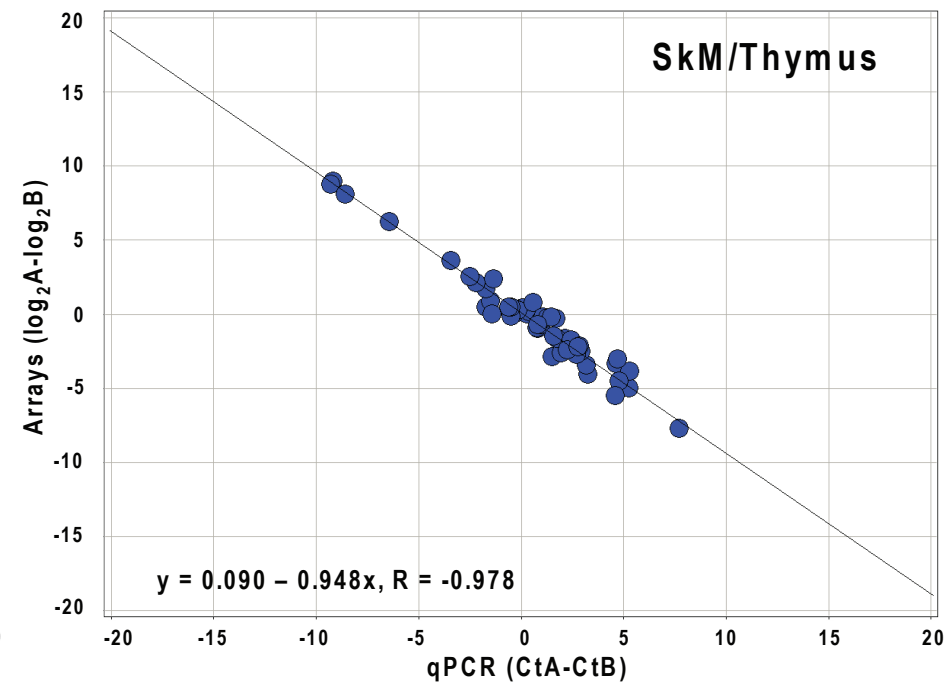

Supplement: Additional File 15 — Comparison of qPCR and microarray miRNA profiling for 60 miRNAs in tissue pairs. Scatter plots for 32 tissue pairs not shown in Figure 3. [file 1472-6750-8-69-S15.pdf]
